# Conformational Analysis of Neutral and Ionic Arginine Forms Using DFT Methods

**DOI:** 10.1021/acsomega.5c01697

**Published:** 2025-06-20

**Authors:** Fulya Çağlar, Gözde Aksoy, Cenk Selçuki

**Affiliations:** † Department of Health Bioinformatics, Institute of Health Sciences, Ege University, 35040 Bornova, Izmir, Turkey; ‡ Faculty of Science, Department of Biochemistry, 37509 Ege University, 35100 Bornova, Izmir, Turkey; § Health Bioinformatics Programme, Graduate School of Health Sciences, Ege University, 35040 Bornova, Izmir, Turkey

## Abstract

Arginine (Arg) is an essential amino acid with a side chain that contains a positively charged group. Arg plays a pivotal role in maintaining the overall charge balance of a protein due to the presence of the guanidino group as its side chain. Consequently, Arg has the capacity to influence various functional characteristics of proteins, encompassing aspects such as folding, solubility, and aggregation. Moreover, the ionic forms of this amino acid can be deemed to be critical determinants in the formation of molecular interactions between proteins and organic compounds. The versatile properties of Arg encompass a broad spectrum of effects, ranging from cellular-level biochemical events to the overall biological functions of the organism. Intriguingly, our knowledge remains incomplete regarding the exhaustive characterization of all potential forms of Arg. This gap in systematic examination within the literature underscores the pressing need for further research in order to comprehensively obtain a clear understanding of the role of Arg within biological systems. The current study aims to investigate all possible conformers of various Arg forms using density functional theory (DFT) in both aqueous and gas phases. First, all possible initial structures for each ionic and neutral Arg form were obtained by conformational analysis using Spartan’16 software. Second, all obtained structures were optimized by DFT using ωB97XD and B3LYP functionals in combination with the 6-311++G­(d,p) basis set as implemented in Gaussian09 software. The two most stable configurations from the optimized geometries were subjected to a reoptimization process using the MP2/6-311++G­(d,p) method to verify their structural integrity. The minimum nature of the optimized structures was verified by frequency analysis performed at both calculation levels. We compared the optimization results of the isoelectronic species in terms of their structures and electronic energies. To the best of our knowledge, these conformations of cationic Arg have not been previously reported in the literature. Also, our calculations have shown that some of the zwitterionic and cationic forms are not stable and are converted to other stable forms through hydrogen transfer from the guanidine group to the α-amino group after optimization by both DFT functionals. According to the results of this study, new stable conformers for Arg were identified in both vacuum and aqueous environments through the applied DFT functionals.

## Graphical abstract

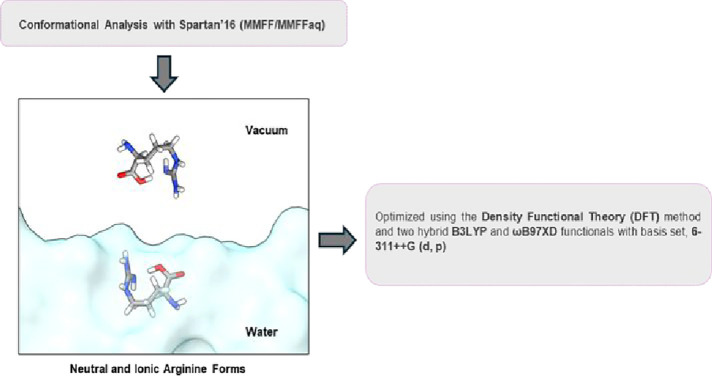

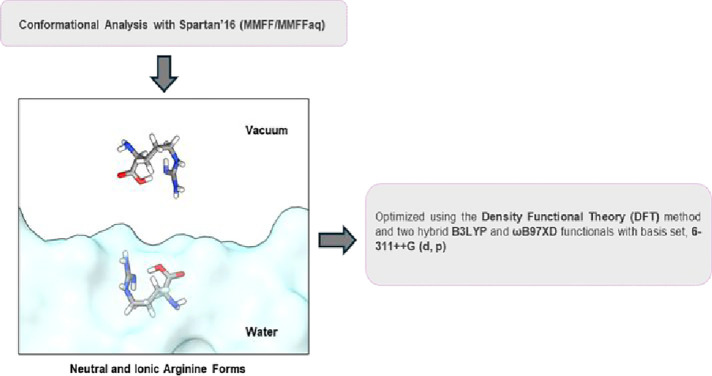

## 1 Introduction

A central α-carbon atom is a common feature of all amino acids, to which both an amino group (NH_2_) and a carboxyl group (COOH) are attached. The α-carbon atom typically forms a bond with either a hydrogen (H) atom or an R group, thus conferring its distinctive feature to the amino acid.^1^

**Figure 1:**
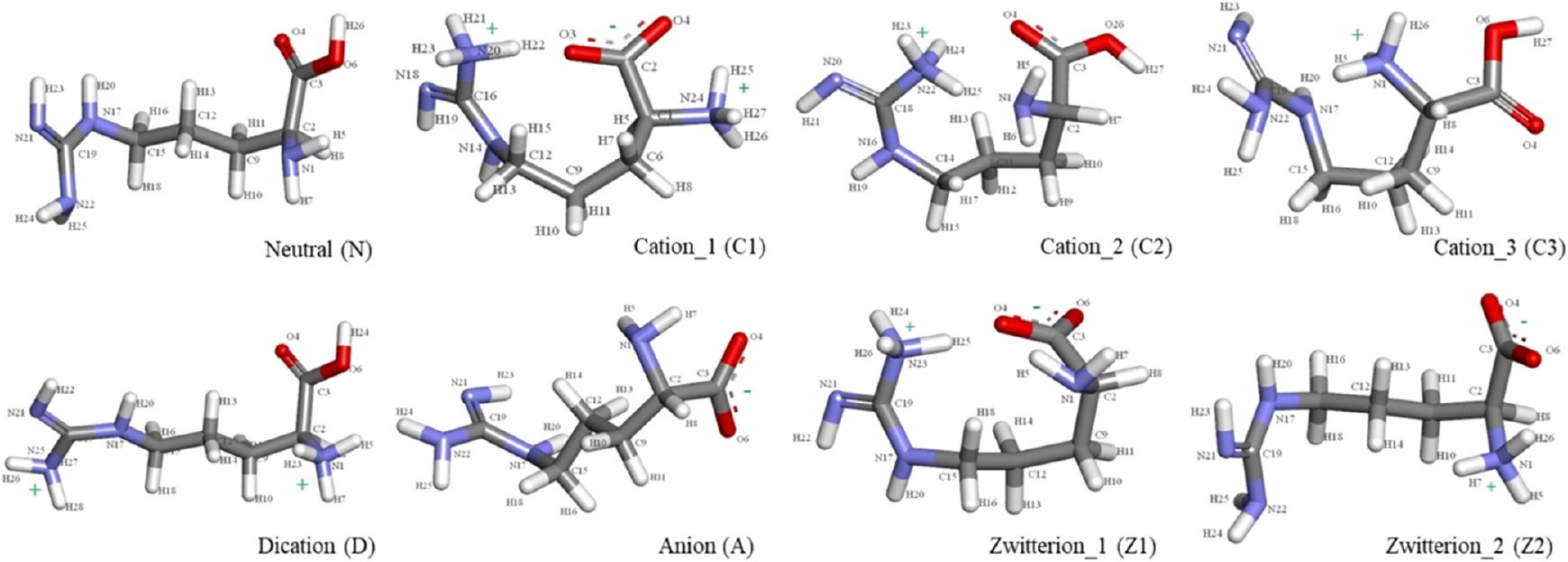
Eight possible geometries of Arg forms.

**Table 1:** Numbers of Initial and Optimized Conformers Were Included in the Study

arginine structure name	DFT method	number of initial structures	number of optimized conformers
AW	ωb97xd	449	329
	b3lyp		327
DW	ωb97xd	36	35
	b3lyp		30
C1W	ωb97xd	30	29
	b3lyp		27
C2W	ωb97xd	222	186
	b3lyp		200
C3W	ωb97xd	35	22
	b3lyp		30
NW	ωb97xd	1190	979
	b3lyp		1021
NV	ωb97xd		1151
	b3lyp		1152
Z1W	ωb97xd	102	85
	b3lyp		101
Z1V	ωb97xd		97
	b3lyp		102
Z2W	ωb97xd	48	45
	b3lyp		43
Z2V	ωb97xd		42
	b3lyp		47

**Figure 2:**
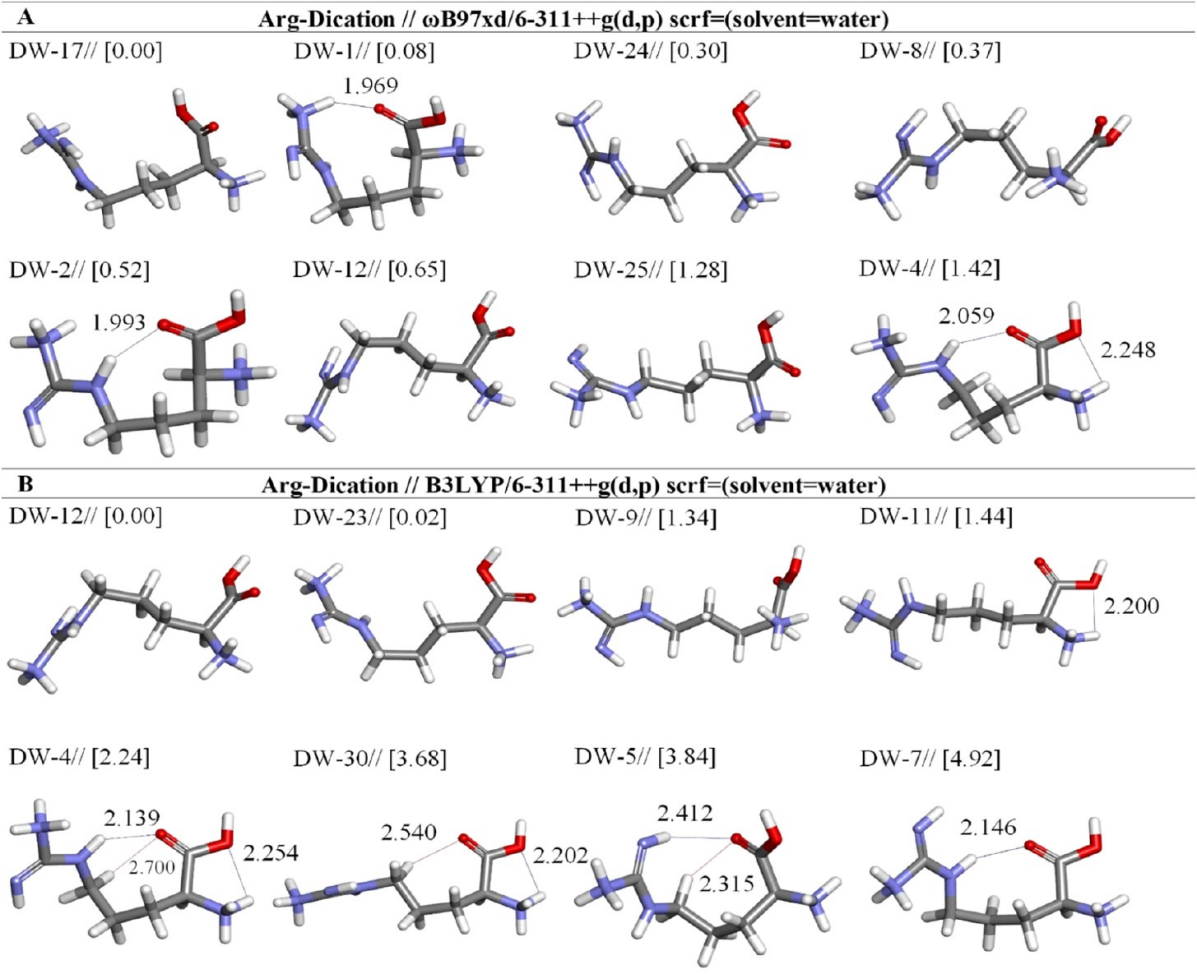
Optimized dication arginine conformers in aqueous by two different DFT methods at the (A) ωb97XD and (B) B3LYP/6-311++G** levels. Distances are given in Å.

**Table 2:** Calculated Total Electronic Energies (*E*_tot_, Hartree), Zero-Point Vibrational Energy Correction (*E* + ZPE, Hartree), Dipole Moment (Debye), and Relative Energies (Δ*E*_rel_, kcal/mol) Using the DFT Method at the B3LYP and ωb97XD Levels of Theory for Dication Species of Arginine

opt. conformer structure	*E*_tot_ (Hartree)^a^	μ (Debye)	*E* + ZPE (Hartree)^a^	Δ*E*_rel_ (kcal mol^–1^)	hydrogen bond	X···H^b^ distance (Å)	%
Arg-Dication-ωb97xd/6-311++g(d,p) (solvent = water)							
DW-17	–607.4413	8.81	–607.1862	0.00			25.25
DW-1	–607.4412	12.06	–607.1860	0.08	:H27-:O4	1.969	22.06
DW-24	–607.4409	4.75	–607.1860	0.30			15.22
DW-8	–607.4407	13.48	–607.1858	0.37			13.52
DW-2	–607.4405	9.46	–607.1855	0.53	:H20-:O4	1.993	10.32
DW-12	–607.4403	14.13	–607.1847	0.65			8.43
DW-25	–607.4393	10.65	–607.1842	1.28			2.91
DW-4	–607.4391	8.29	–607.1837	1.42	:H20-:O4	2.059	2.30
					:H5-:O6	2.248	
Arg-Dication-b3lyp/6-311++g(d,p) (solvent = water)							
DW-12	–607.6249	13.88	–607.3736	0.00			45.92
DW-23	–607.6251	6.89	–607.3736	0.02			44.06
DW-9	–607.6225	10.84	–607.3715	1.34			4.78
DW-11	–607.6227	3.45	–607.3713	1.44	:H5-:O6	2.200	4.02
DW-4	–607.6219	8.16	–607.3700	2.24	:H20-:O4	2.139	1.05
					:H16-:O4	2.700	
					:H5-:O6	2.254	
DW-30	–607.6194	8.99	–607.3677	3.68	:H18-:O4	2.540	0.09
					:H5-:O6	2.202	
DW-5	–607.6189	10.74	–607.3675	3.84	:H22-:O4	2.412	0.07
					:H16-:O4	2.315	
DW-7	–607.6175	10.25	–607.3658	4.92	:H20-:O4	2.146	0.01

a Hartree = 627.503 kcal/mol.

b X acceptor (N or O).

**Figure 3:**
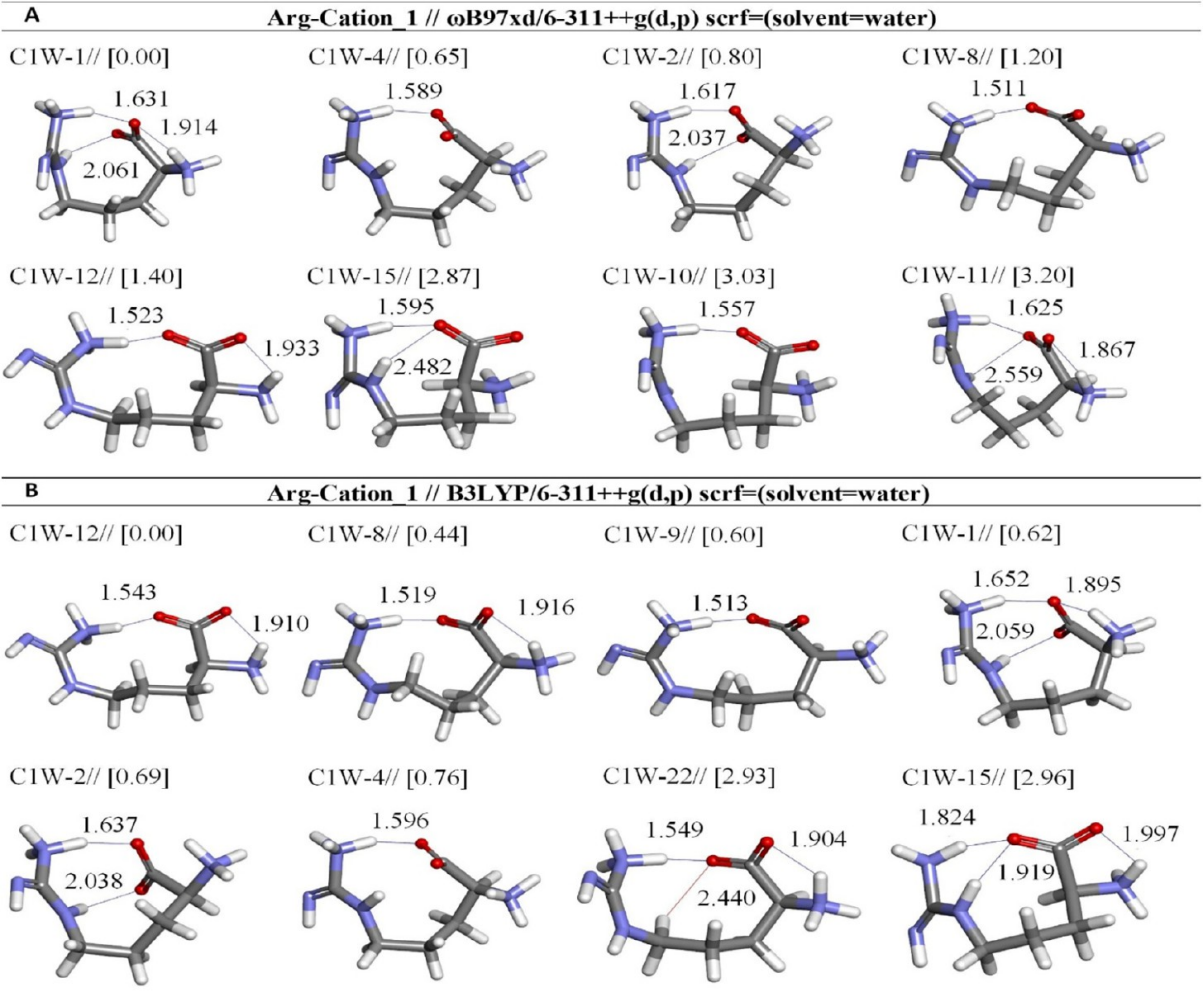
Optimized results of cation-1 Arg conformers in aqueous by two different DFT methods at the (A) ωb97XD and (B) B3LYP/6-311++G** levels. Distances are given in Å.

**Table 3:** Calculated Total Electronic Energies (*E*_tot_, Hartree), Zero-Point Vibrational Energy Correction (*E* + ZPE, Hartree), Dipole Moment (Debye), and Relative Energies (Δ*E*_rel_, kcal/mol) Using the DFT Method at the B3LYP and ωb97XD Levels of Theory for Cation-1 Species of Arginine

opt. conformer structure	*E*_tot_ (Hartree)^a^	μ (Debye)	*E* + ZPE (Hartree)^a^	Δ*E*_rel_ (kcal mol^–1^)	hydrogen bond	X···H^b^ distance (Å)	(%)
Arg-Cation1-ωb97xd/6-311++g(d,p) (solvent = water)							
C1W-1	–607.0122	6.49	–606.7704	0.00	:H22-:O4	1.631	54.43
					:H17-:O3	2.061	
					:H27-:O4	1.914	
C1W-4	–607.0111	8.08	–606.7696	0.65	:H21-:O3	1.589	18.27
C1W-2	–607.0109	7.10	–606.7693	0.80	:H22-:O4	1.617	14.05
					:H17-:O3	2.037	
C1W-8	–607.0102	9.08	–606.7692	1.20	:H21-:O3	1.511	7.13
C1W-12	–607.0099	12.71	–606.7689	1.40	:H22-:O3	1.523	5.12
					:H27-:O4	1.933	
C1W-15	–607.0076	7.87	–606.7666	2.87	:H23-:O3	1.595	0.43
					:H17-:O3	2.482	
C1W-10	–607.0073	13.90	–606.7663	3.03	:H21-:O3	1.557	0.33
C1W-11	–607.0071	7.26	–606.7658	3.20	:H21-:O3	1.625	0.25
					:H25-:O4	1.867	
					:H17-:O3	2.559	
Arg-Cation1-b3lyp/6-311++g(d,p) (solvent = water)							
C1W-12	–607.1956	12.46	–606.9577	0.00	:H22-:O3	1.543	35.89
					:H27-:O4	1.933	
C1W-8	–607.1948	9.05	–606.9570	0.44	:H21-:O3	1.519	16.97
					:H26-:O4	1.916	
C1W-9	–607.1940	8.90	–606.9568	0.60	:H23-:O3	1.513	12.95
C1W-1	–607.1953	6.62	–606.9567	0.62	:H22-:O4	1.652	12.55
					:H17-:O3	2.059	
					:H27-:O4	1.895	
C1W-2	–607.1947	7.22	–606.9566	0.69	:H22-:O4	1.637	11.19
					:H17-:O3	2.038	
C1W-4	–607.1946	8.03	–606.9565	0.76	:H21-:O3	1.596	9.95
C1W-22	–607.1908	8.92	–606.9531	2.93	:H22-:O3	1.549	0.26
					:H13-:O3	2.440	
					:H25-:O4	1.904	
C1W-15	–607.1917	8.06	–606.9530	2.96	:H23-:O3	1.824	0.24
					:H17-:O3	1.919	
					:H25-:O4	1.997	

a Hartree = 627.503 kcal/mol.

b X acceptor (N or O).

**Figure 4:**
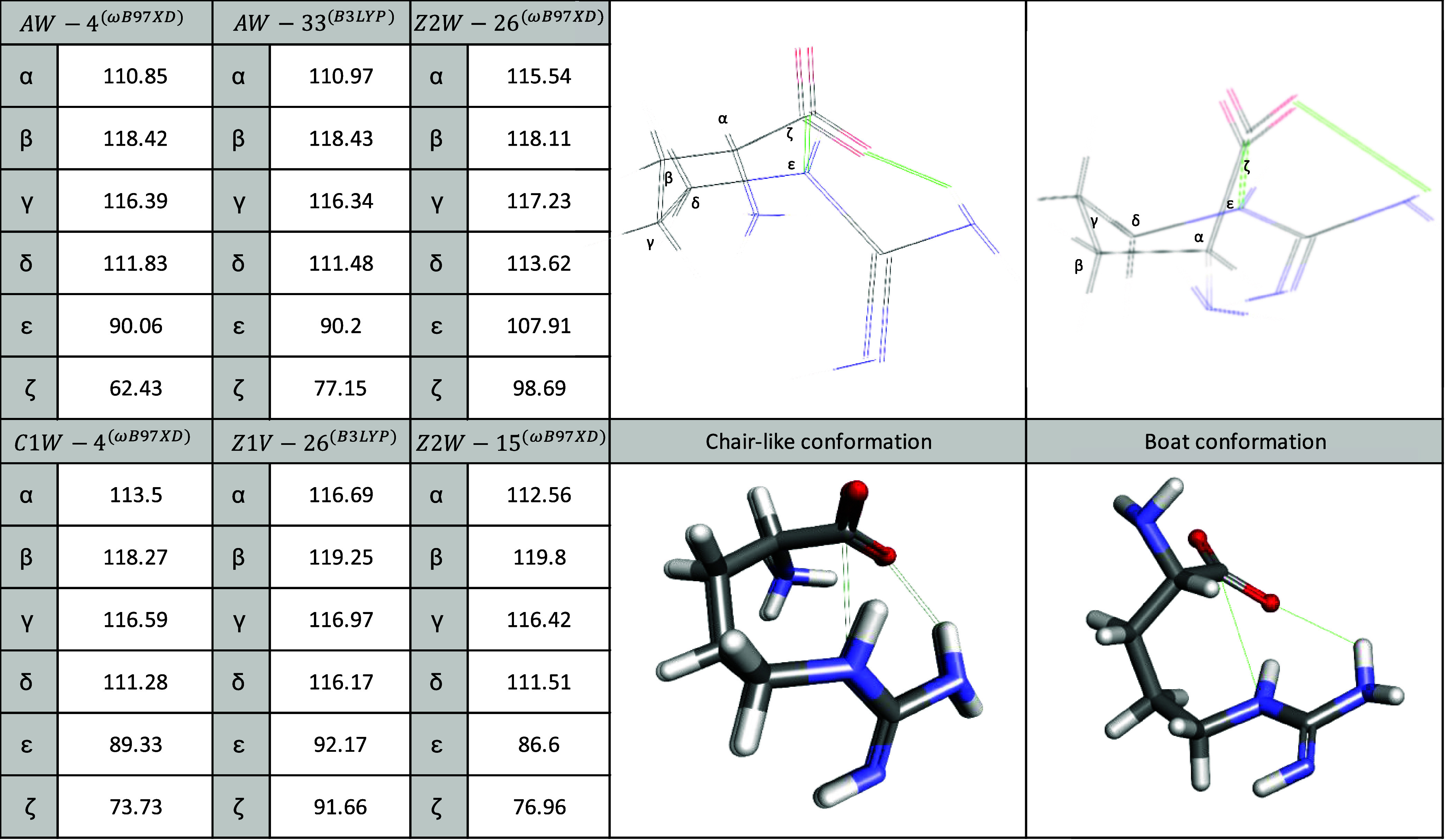
Angles in degrees of chair-like and boat-like conformers of Arg. Examples for the chair-like conformation of Z1V and C1W and boat-like of AW.

**Figure 5:**
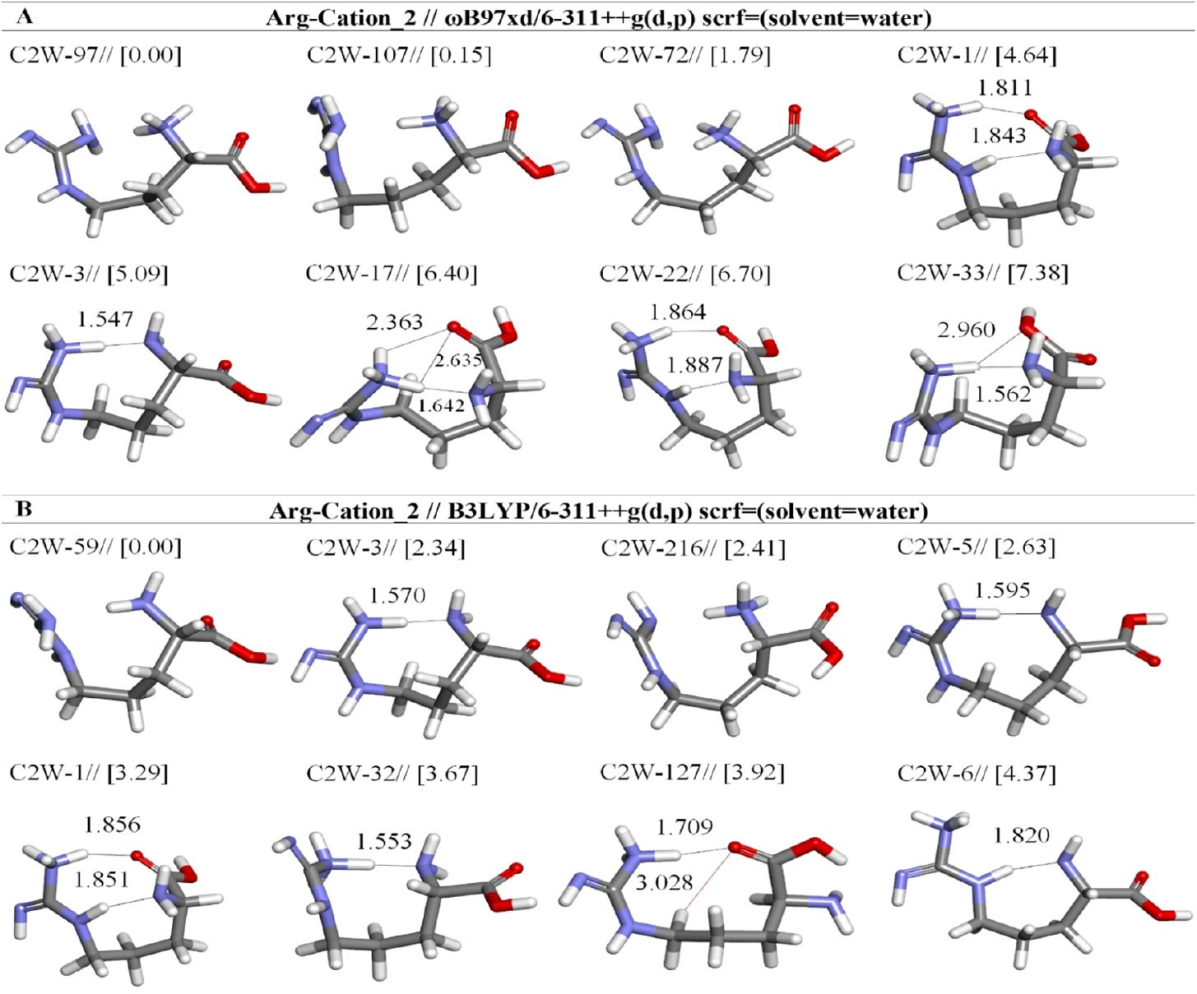
Optimized results of cation-2 Arg conformers in aqueous by two different DFT methods at the (A) ωb97XD and (B) B3LYP/6-311++G** levels. Distances are given in Å.

**Table 4:** Calculated Total Electronic Energies (*E*_tot_, Hartree), Zero-Point Vibrational Energy Correction (*E* + ZPE, Hartree), Dipole Moment (Debye), and Relative Energies (Δ*E*_rel_, kcal/mol) Using the DFT Method at the B3LYP and ωb97XD Levels of Theory for Cation-2 Species of Arginine

opt. conformer structure	*E*_tot_ (Hartree)^a^	μ (Debye)	*E* + ZPE (Hartree)^a^	Δ*E*_rel_ (kcal mol^–1^)	hydrogen bond	X···H^b^ distance (Å)	(%)
Arg-Cation 2-ωb97xd/6-311++g(d,p) (solvent = water)							
C2W-97	–607.0166	3.94	–606.7758	0.00			54.94
C2W-107	–607.0164	6.28	–606.7752	0.15			42.36
C2W-72	–607.0138	5.00	–606.7729	1.79			2.67
C2W-1	–607.0092	5.03	–606.7698	4.64	:H25-:O4	1.811	0.02
					:H19-:N1	1.843	
C2W-3	–607.0085	3.68	–606.7698	5.09	:H23-:N1	1.547	0.01
C2W-17	–607.0064	1.69	–606.7671	6.40	:H25-:O4	2.363	0.00
					:H23-:O4	2.635	
					:H23-:N1	1.642	
C2W-22	–607.0060	4.95	–606.7660	6.70	:H25-:O4	1.864	0.00
					:H19-:N1	1.887	
C2W-33	–607.0054	4.99	–606.7657	7.08	:H23-:O26	2.960	0.00
					:H23-:N1	1.562	
Arg-Cation 2-b3lyp/6-311++g(d,p) (solvent = water)							
C2W-59	–607.1979	5.24	–606.9606	0.00			94.69
C2W-3	–607.1928	3.65	–606.9569	2.34	:H23-:N1	1.570	1.82
C2W-216	–607.1944	4.16	–606.9568	2.41			1.62
C2W-5	–607.1920	6.35	–606.9564	2.63	:H25-:N1	1.595	1.12
C2W-1	–607.1925	4.88	–606.9554	3.29	:H25-:O4	1.856	0.37
					:H19-:N1	1.851	
C2W-32	–607.1900	6.16	–606.9548	3.67	:H23-:N1	1.553	0.19
C2W-127	–607.1917	7.31	–606.9544	3.92	:H23-:O4	1.709	0.13
					:H15-:O4	3.028	
C2W-6	–607.1896	9.19	–606.9536	4.37	:H19-:N1	1.820	0.06

a Hartree = 627.503 kcal/mol.

b X acceptor (N or O).

**Table 5:** Calculated Total Electronic Energies (*E*_tot_, Hartree), Zero-Point Vibrational Energy Correction (*E* + ZPE, Hartree), Dipole Moment (Debye), and Relative Energies (Δ*E*_rel_, kcal/mol) Using the DFT Method at the B3LYP and ωb97XD Levels of Theory for Cation-3 Species of Arginine

opt. conformer structure	*E*_tot_ (Hartree)^a^	μ (Debye)	*E* + ZPE (Hartree)^a^	Δ*E*_rel_ (kcal mol^–1^)	hydrogen bond	X···H^b^ distance (Å)	(%)
Arg-Cation 3-ωb97xd/6-311++g(d,p) (solvent = water)							
C3W-19	–607.0466	10.33	–606.8078	0.00	:H7-:N1	1.839	69.96
C3W-17	–607.0452	7.91	–606.8071	0.87	:H7-:N1	1.898	16.05
C3W-13	–607.0443	11.24	–606.8056	1.41	:H5-:N1	1.847	6.51
C3W-26	–607.0436	10.20	–606.8052	1.85	:H7-:N1	1.895	3.08
C3W-8	–607.0433	12.13	–606.8048	2.02	:H5-:N1	1.848	2.30
C3W-10	–607.0432	9.76	–606.8043	2.09	:H7-:N1	1.844	2.06
C3W-14	–607.0393	7.99	–606.8004	4.52	:H7-:O6	2.922	0.03
					:H7-:N1	1.836	
C3W-4	–607.0278	3.76	–606.7886	11.78	:H5-:N21	1.571	0.00
Arg-Cation 3-b3lyp/6-311++g(d,p) (solvent = water)							
C3W-7	–607.2305	10.43	–606.9954	0.00	:H5-:N1	1.859	54.97
C3W-6	–607.2294	12.75	–606.9943	0.70	:H5-:N1	1.951	16.86
C3W-12	–607.2291	8.79	–606.9939	0.94	:H7-:N1	1.944	11.27
C3W-8	–607.2290	12.29	–606.9938	1.01	:H5-:N1	1.880	10.05
C3W-13	–607.2282	11.37	–606.9930	1.51	:H5-:N1	1.873	4.30
C3W-26	–607.2276	10.46	–606.9925	1.83	:H7-:N1	1.946	2.51
C3W-4	–607.2245	11.00	–606.9884	4.37	:H5-:N1	1.917	0.03
					:H8-:N17	2.672	
C3W-30	–607.2209	5.71	–606.9852	6.43	:H7-:N21	1.521	0.00

a Hartree = 627.503 kcal/mol.

b X acceptor (N or O).

**Figure 6:**
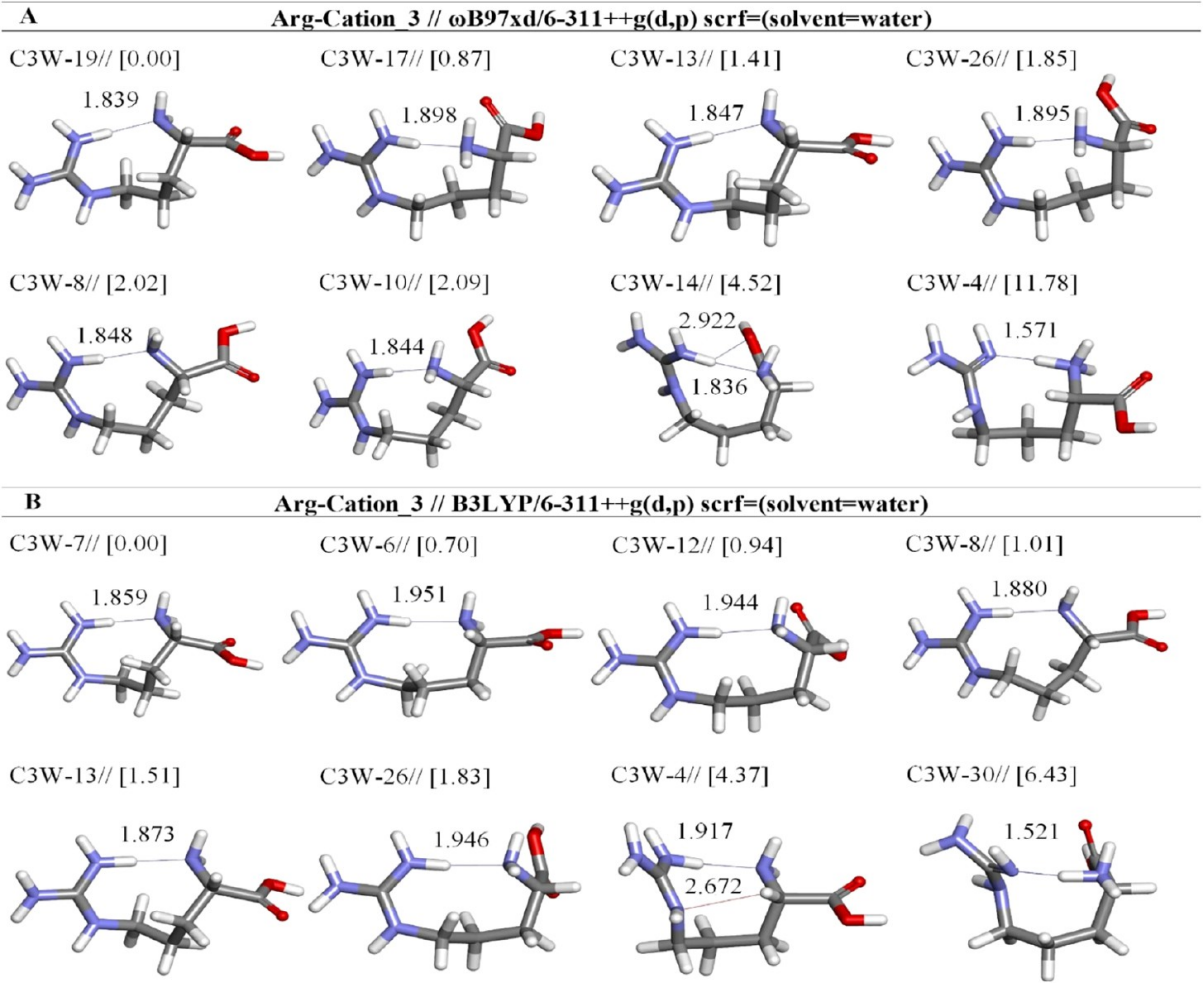
Optimized results of cation-3 Arg conformers in aqueous by two different DFT methods at the (A) ωb97XD and (B) B3LYP/6-311++G** levels. Distances are given in Å.

The guanidinium group is a nitrogenous analogue of carbonic acid. It has been widely described in molecular recognition occurring by hydrogen bonding and/or electrostatic and cation-π interactions. This moiety is found in the side chain of Arg. Unlike other amino acids, Arg has a positive charge at neutral pH due to the presence of this group in the side chain. This implies that even under physiological conditions, the side chain maintains its own charge while residing in the hydrophobic interior region of a folded protein.^2–5^ Recent studies have emphasized the effective preservation of the positive charge of Arg amino acid due to its high intrinsic dissociation constant (p*K*_a_ ≈ 13.8 ± 0.1).^6^ From this perspective, Arg plays a crucial role in maintaining the charge balance of a protein and also resides at the central positions of the functional domains for many reactive enzymes. Additionally, it significantly contributes to processes such as protein folding,^7^ solubility,^8^ and aggregation.^9^

The zwitterionic form of Arg plays a significant role in biochemical processes by stabilizing molecular structures in water.^10,11^ The zwitterionic structures have zero total charge but contain both positively and negatively charged centers, in contrast to the neutral structure. In an aqueous solution, amino acids achieve this equilibrium by deprotonation of the carboxyl group (CO^2–^) and protonation of amino groups (H_3_N^+^). Although zwitterionic forms of amino acids are stable in aqueous solutions, it has been observed that they cannot maintain their stabilities in the gas phase and convert back to the neutral form. This behavior indicates that the zwitterionic charge separation in the gas phase cannot be stabilized by the environment.^12,13^ The guanidinium group of Arg has a high proton affinity. Therefore, especially in the gas phase, zwitterionic structures have attracted significant attention in both computational and experimental studies, and it has been observed that the zwitterionic form is more stable compared to other forms.^14^ However, experimental evidence has shown that Arg cannot preserve this form in the gas phase. Theoretically, Arg residues that are in the hydrophobic internal positions in the protein are charged positively in an aqueous solution due to the presence of the same guanidine groups.^15–19^ However, experimental observations of this theory are very rare. Therefore, supporting experimental data through computational chemistry methods can be of significant importance and can play a crucial role in contributing to experimental findings. In this context, studies on the structural transitions and stability properties of Arg in the gas phase have yielded significant findings.^3,11,12,17,18,20^

Experimental spectrum data, obtained by Chapo and colleagues using high-resolution laser spectroscopy (IR-CRLAS),^20^ have provided valuable insights into the molecular structure of Arg in the gas phase. Subsequently, Rak and his team conducted a comprehensive analysis of these experimental data using ab initio calculations.^18^ The calculations employed B3LYP/6-31++G** and MP2/6-31++G** levels and revealed the transformation of Arg from the zwitterion form to the neutral form.

However, conflicting results have arisen from another study. Williams and colleagues conducted a similar investigation using a different density functional theory (DFT) method.^21^ In this study, it was observed that the zwitterionic forms of Arg are more stable than the neutral form in the gas phase, which contradicts the previous findings. These contradictory outcomes underscore the complexity and challenge of understanding the behavior of Arg in the gas phase. To gain further insights and clarify these conflicting results, we required additional ab initio studies are required.

Determining the global minimum of Arg through computational methods is a complex endeavor compared to experimental studies due to the presence of multiple proton-donating (OH and NH) and proton-accepting (N and O) groups within its molecular structure. Consequently, calculating the most stable Arg conformers requires the consideration of intricate interactions and multiple conformations among these groups. For this reason, methods including strong force fields, minimum energy search algorithms, and large basis sets should be preferred in computational studies.^22^ Density functional theory (DFT) is a quantum mechanics method that is most commonly used to calculate the ground-state energy of a given system and to predict the electronic structure of molecules and solid.^23^ In most computational approaches, optimizations of Arg forms have been generally carried out with the B3LYP functional in combination with various basis sets.^23–25^ However, some research groups argue that the DFT method cannot accurately predict the structure for zwitterionic forms of Arg due to its low specificity and sensitivity. So, we have included not only those hybrids functional but also the ωB97XD hybrid functional which takes the weak interactions into account in our work.^26^

Previous studies on Arg have focused on only the neutral and zwitterionic forms in the gas phase. However, a systematic approach for all possible forms of Arg has not yet been reported in the literature yet. Thus, we aimed to identify the structure, geometry, and minimum energy structures of all possible Arg forms in both aqueous and gas media. First, we determined all possible initial structures for each Arg form by conformational analysis using Spartan’16 software.^27^ Next, all obtained structures were optimized by DFT method using ωB97XD and B3LYP functionals in combination with the 6-311++G­(d,p) basis set as implemented in Gaussian09 software.^28^ The minimum nature of the optimized structures was verified by a frequency analysis. In this study, detailed analysis of the structure, energy, and electronic properties of neutral and ionic Arg species has been conducted.

## 2 Computational Methods

The ionic forms of Arg (dication, cation, and anion) were exclusively examined in aqueous phase (water = w), while the neutral forms (zwitterion and neutral) were investigated both in the aqueous solution and in gas phases (vacuum = v) (Figure 1).

All initial geometries were determined with Spartan’16 ^27^ using the Merck molecular force field (MMFF/MMFFaq). In modeling, the conformers were created by rotating the torsion angles of the C_∝__C_β_, C_β__C_γ_, C_∝__N, C_∝__COOH, C_δ__N and C_δ‑NH__C single bonds by 60° (360°/6-fold) in the initial geometries of Arg structures. Considering the electrostatic properties of Arg, selected structures were additionally optimized using the Density Functional Theory (DFT) method and two hybrid B3LYP and ωB97XD functionals with basis set, 6-311++G (d,p) using Gaussian09 software.^28^ In addition, further optimizations utilizing the MP2 method were conducted to confirm the stability of the most stable structures. To account for solvent effects in calculations involving the aqueous phase, the Polarizable Continuum Model (PCM) was utilized as the implicit model for solvation.

Utilizing Boltzmann distribution principles at a temperature of 298.15 K, we determined the arrangement of the ten most energetically stable conformers from the entire set of conformer structures. The distribution of conformers was established based on the principles of the Boltzmann distribution, as outlined in eq 1 presented below:

1 $$\frac{N_{i}}{N_{0}}=-e^{\left[\left(E_{i}-E_{0}\right)/k_{B}T\right]}$$

Relative energy (Δ*E*_rel_ - kcal/mol) values were calculated for all possible conformers of each Arg form. Relative energy represents the calculated energy difference for a molecule or structure. The structure with the most negative electronic energy is compared to other structures using it as a reference. This reference energy level is set at 0.00 kcal/mol. Therefore, the structure with the most negative energy is considered more stable when compared to other structures, and its relative energy is calculated as 0.00 kcal/mol. In the above equation, *N_i_*/*N*_0_ is the ratio of the *i*th order high-energy conformation over the ground state. T is the temperature in Kelvin, *k*_B_ is the Boltzmann’s constant and *E*_*i*_ is the total electronic energy of a molecular configuration, *E*_*i*_ – *E*_0_ is the energy difference between the energy in the ground state and the conformer energies. From the present calculations of all conformers, the total electronic energy (*E*_tot_ - Hartree), dipole moment (μ - Debye), and zero-point vibrational correction energies (ZPE - Hartree) were reported. Molecular modeling of all of the Arg structures and hydrogen bonding representations were created using the Discovery Studio Visualizer-2020 software.^29^

The various forms of Arg are commonly denoted by different abbreviations in the literature. However, due to the distinct initial structures used in this study, a new nomenclature system was used. To enhance the comprehensibility of our work, we reconfigured the notation for Arg conformers by examining the net charge distribution within the Arg molecule. Throughout our study, we chose to use the abbreviations N, Z (Z1 and Z2), A, D, and C (C1, C2, and C3) instead of the conventional nomenclature for the neutral, zwitterionic, anionic, dicationic, and cationic forms, respectively.

The abbreviations of gas phase = vacuum (V) and aqueous (W) were used preceding the structure name. Since there are too many optimized structures, only the eight most stable conformers for each Arg form were given in the text.

## 3 Results and Discussion

Molecular mechanics methods use force fields to obtain information about the potential energy surface of a molecular system. These force fields can be parametrized to fit experimental or high-level computational data for small to large biological systems. Finding new conformers from the initial structures often requires high-level MM calculations. In addition, the reliability of the force fields used is important for high-parameter optimizations, as weak initial geometries may not lead to global minima. Thus, MMFF/MMFFaq force fields were preferred for the conformational analysis of all Arg forms in both aqueous and gas media.^30,31^ Full geometry optimization and electronic structures of all conformers of Arg were performed with density functional theory at the B3LYP/6-311++G­(d,p) and ωb97XD/6-311++G­(d,p) levels. Table 1 shows the numbers of initial and optimized conformers. Additionally, to corroborate the DFT outcomes, MP2 calculations were executed on the conformers with the lowest energy, lending further credence to the structural integrity of the Arg conformers. The comparison of optimized geometries from B3LYP/6-311++G­(d,p) and ωB97XD/6-311++G­(d,p) with those from MP2/6-311++G­(d,p) showed no substantial structural changes, reflecting a reliable agreement between the various computational approaches (Figures S1 and S2).

### 3.1 Optimized Structure of Arginine in an Aqueous Solution

The ionic (anionic, cationic, and dicationic), zwitterionic, and neutral forms of Arg were investigated in terms of structural and electronic properties.

#### 3.1.1 Dicationic Arg Conformers

At the B3LYP/6-311++G and ωb97XD/6-311++G levels, a total of 75 local minima were identified. When the rankings of electronic energies are examined, structures DW-12 (*E*_tot_ = −607.6249 hartree) and DW-17 (*E*_tot_ = −607.4413 hartree) emerge as the most stable conformers, respectively. These structures have been optimized using the B3LYP and ωb97XD methods, respectively (Table 2 and Figure 2).

The most stable dicationic Arg is DW-12 with B3LYP. However, according to the optimization results obtained using the ωb97XD functional, the most stable structure was identified as the DW-17, and it was determined to be 0.65 kcal/mol more stable than the DW-12 which ranked sixth after optimization (Figure 2 and Table 2). The dipole moment serves as a crucial metric for assessing the polarity and charge distribution of a molecule. When we compare the dipole moments of DW-17 and DW-12 following optimization at the ωb97XD level, it becomes evident that DW-12 exhibits a higher dipole moment. This signifies that the molecule is more polar, with a more distinct separation of positive and negative charges. Changes were observed in the positions of the η1 (NH) and η2 (NH3) groups bound to Cζ. The positioning of these groups varies within the 3D structures of conformers compared to the NH3 group bound to Cα. In the DW-12 conformation, these groups align in the same direction, whereas in the DW-17 conformation, they adopt opposite orientations.

These structural distinctions arise from subtle alterations in the molecule’s three-dimensional arrangement. Such changes in atom positions can exert an influence on the chemical properties of the molecule. These structural variations hold the potential to significantly impact molecular interactions, reactivity, and molecular characteristics, potentially altering the course of specific chemical reactions. Analyses of this nature serve as crucial research tools for comprehending phenomena at the molecular level.

The energy difference between DW-17 and DW-1, as examined using the ωb97XD method, indicates only a very low value, approximately 0.08 kcal mol-1 while the electronic energies of both conformations are quite similar; significant changes have been observed in their structural geometries. In the geometry of the DW-1, hydrogen bonds (N–H(27)···O(4)=C_α‑COOH_, 1.969 Å) are formed (Figure 2a and Table 2). This situation may be attributed to the proximity of the carboxyl and amino groups to each other.

The stability order has been found as DW-17 > DW-25 > DW-4 in our optimization results for dication forms of Arg at ωb97XD/6-311++G­(d,p) level (Table 2 and Figure 2a). The DW-4 is less stable with respect to DW-17 by 1.42 kcal mol^–1^, It has been observed that both N–H(20)···O(4)=C_α‑COOH_ (2.059 Å) hydrogen bond and N_α‑NH_-H­(5)···O­(6)=C_α‑COOH_ (2.248 Å) hydrogen bond coexisted in intramolecular intermediates, unlike other dication conformers (Figure 2). In addition, it was seen that DW-4 is far less stable than DW-12 by 2.24 kcal mol^–1^ in the ωb97XD method, too (Table 2). Looking at intramolecular parameters such as bond lengths and bond types in the geometry of DW-4, it has been observed to include two hydrogen bonds (N–H(20)···O(4)=C_α‑COOH_ (2.139 Å) and N_α‑NH_-H­(5)···O­(6)=C_α‑COOH_ (2.254 Å)), and one nonclassical (weak) hydrogen bond (C–H(16)···O(4)=C_α‑COOH_ (2.700 Å)) (Figure 2 and Table 2).

DW-9 and DW-11 have higher energies than DW-4. DW-11 is different from DW-9 due to the formed N_α‑NH_-H­(5)···O­(6)=C_α‑COOH_ (2.200 Å) hydrogen bond, and differences in electronic energy between them is less by 0.10 kcal mol^–1^. The DW-5 is less stable with respect to DW-4 by 3.84 kcal mol^–1^. N_ζ‑NH_-H···O=C_α‑COOH_ hydrogen bond is to be expected bond type in other Arg forms which are zwitterionic and neutral.^15,32^

The analysis of free energy values derived from the ωB97XD/6-311++G­(d,p) method reveals that DW-17 is the most stable conformer with a notable occurrence rate of 25.25%. DW-1 is identified as the second most stable structure, boasting an abundance of 22.06%, while DW-24, DW-8, and DW-2 present similar abundance levels of 15.22, 13.52, and 10.32%, respectively. According to the results derived from the B3LYP/6-311++G­(d,p) method, DW-12 shows the most stability, with a percentage of 45.92%, followed by DW-23, which has a stability of 44.06%. The remaining conformers are found in smaller quantities, reflecting their relatively lower stability (Table 2).

#### 3.1.2 Cationic Arg Conformers

The Arg side chain retains its positive charge under all physiological conditions due to the presence of the guanidinium group. In this way, it is highly effective on the properties of proteins such as folding, solubility, and aggregation. Therefore, the cationic form of Arg plays an important role in determining the properties of the molecular interactions between various organic compounds.

In our study, cationic forms of the Arg were obtained from the initial neutral structure by deprotonation and/or protonation. For cationic forms named C1, both the guanidine group and the α-amino group were protonated, and the α-carboxyl group was deprotonated. Unlike C1, in C2, only the guanidine group is protonated, and in C3, only the α-amino group is protonated (Figure 1). The results obtained with the ωb97XD/6-311++G­(d,p) level suggest that the cationic structure C1W-1 (Δ*E*_rel_ = 0.00 kcal/mol) is the most stable, exhibiting significantly higher stability with an abundance of 54.43% compared to other conformers and C1W-4 (Δ*E*_rel_ = 0.65 kcal/mol) the second most stable structures which exhibit abundances of 18.27% (Table 3). The oxygen atom (O4) of the –O=Cα-COO– group forms H-bonds (1.914 Å) with a hydrogen atom H(27) of the α-amino group in C1W-1, but not all in C1W-4 (Figure 3a and Table 3). It is clearly demonstrated that at this level C1W-1 and C1W-4 are very close in energy by 0.65 kcal/mol but C1W-1 is more stable than C1W-12 by 1.40 kcal/mol. However, it was observed that C1W-12 is the most stable structure in B3LYP. In the results obtained using the B3LYP/6-311++G­(d,p) method, the C1W-12 demonstrates the highest stability, being the most stable conformer with an abundance of 35.89%. Following this, the conformers C1W-8, C1W-9, C1W-1, C1W-2, C1W-4, C1W-22, and C1W-15 exhibit lower abundance values of 16.97, 12.95, 12.55, 11.19, 9.95, 0.26, and 0.24%, respectively (Figure 3b and Table 3).

The geometric analyses reveal that six distinct conformers (C1W-1, C1W-4, C1W-2, C1W-8, C1W-12, and C1W-15) exhibit structural similarities in terms of the orientations of the guanidine group, amino group, and carboxylate group, regardless of whether the B3LYP or ωb97XD methods are employed. Furthermore, we found that the values of H-bond lengths determined at ωb97XD are longer compared to those calculated by B3LYP. Additionally, we have observed that C1W-4 forms an open six-membered ring and exhibits a chair-like conformation (as shown in Figure 4).

Within the scope of our study, the initial structure of the C2 group was established by protonation of the amino group in the neutral Arg. However, according to the results of the optimization process, it was observed that proton transfer occurred between the guanidinium and amino groups in stable conformations calculated using the ωb97XD functional. This observation provided an opportunity to investigate the probability of proton transfer at the molecular level and to evaluate the energetic outcomes of this process in more detail (Figure 5).^33^ The proton is transferred not from the carboxylic acid (−OH) group but from the N(22)-H group. The stable structures of C2W-97_[0.00]_^ωb97XD/6‑311++G(d,p)^ and C2W-59_[0.00]_^B3LYP/6‑311++G(d,p)^ were found to lack intramolecular hydrogen bonds, unlike other conformers (Figure 5 and Table 4).

The C2W-107 is the second most stable conformer with respect to C2W-97 by 0.15 kcal/mol at ωb97XD level, while C2W-3 is second stable by 2.34 kcal/mol at B3LYP level, but it has an intramolecular H-bond (C_ζ‑NH_-H­(23)···N­(1)-C_α‑NH_) different from C2W-107. The free energy values calculated using the ωB97XD/6-311++G­(d,p) method indicate that C2W-97 is the most stable structure, exhibiting a high stability with an abundance of 54.94%. This is followed by C2W-107, which shows an abundance of 42.36%. On the other hand, the C2W-59 stands out as the leading structural form, showcasing a significant prevalence of 94.69% based on the findings from the B3LYP/6-311++G­(d,p) approach. Other conformers, by comparison, reveal significantly lower abundance rates, such as C2W-3 (1.82%), C2W-216 (1.62%), C2W-5 (1.12%), C2W-1 (0.37%), C2W-32 (0.19%), and C2W-127 (0.13%). This considerable difference in abundance underscores the unique stability of C2W-59, suggesting its potential reactivity and its effect on molecular interactions. The significant influence of C2W-59 suggests it could be vital in influencing the system’s behavior being examined (Table 4).

When C3W-4 is the eighth lowest energy structure at the ωb97XD method, it is the seventh lowest energy structure at the B3LYP method. It is less stable than C3W-19 and C3W-7 by 11.78 and 4.37 kcal/mol, respectively (Table 5). In our study, a significant observation is that low-energy C3W-4 in the C3 group, particularly at the ωb97XD/6-311++G­(d,p) level, maintains the protonation of guanidium and does not convert to a neutral structure, unlike other conformers. However, the results obtained with the B3LYP method do not reflect this situation for the C3W-4. When optimized with the B3LYP method, C3W-4 undergoes proton transfer by losing its proton from the N(1)H group, resulting in a neutral structure. A similar situation is observed for B3LYP-optimized C3W-30, where the positive charge on the guanidinium group is retained after optimization.

The calculations of free energy utilizing the ωB97XD/6-311++G­(d,p) method reveal that C3W-19 stands out as the most stable conformer, demonstrating remarkable stability with a prevalence of 69.96%. Following this, C3W-17 demonstrates a notable abundance of 16.05%. The other conformers, which include C3W-13, C3W-26, C3W-8, C3W-10, C3W-14, and C3W-4, show reduced abundance levels of 6.51, 3.08, 2.30, 2.06, 0.03, and 0.00%, in that order. The analysis conducted using the B3LYP/6-311++G­(d,p) method suggests that the C3W-7 is the most stable, with its occurrence reaching 54.97%, making it the most frequently observed conformer (Table 5).

This observation suggests that low-energy structures in the C3 group have the amino group positioned farther from the guanidinium group, and this arrangement may influence the proton transfer process. In conclusion, it was observed that, following the optimization in aqueous solution, all conformers in the C3 group, except for C3W-4 at the ωb97XD level, and C3W-30 at the B3LYP level, were observed to convert from their cationic properties to a neutral form (Figure 6). This may be due to the fact that charge distribution within the cation by balancing would like to become more stable in aqueous solution. For the C3W-4 geometry, the basic site N(1) and the basic site N(21) are connected by a hydrogen bond in both DFT methods. But N(21) acts as a proton acceptor in C3W-4_[11.78]_^ωb97XD/6‑311++G(d,p)^ and as a proton donor in C3W-4_[4.37]_^B3LYP/6‑311++G(d,p)^ C3W-4 structure is characterized by a C_ζ‑NH_-H­(5)···N­(1)-C_α‑NH_ H-bond with a distance of 1.917 Å in B3LYP (Figure 6a) and a shorter but significantly bent C_ζ‑NH_-H­(5)···N­(21)-C_α‑NH_ bond has a distance of 1.517Å in other (Figure 6b).

#### 3.1.3 Zwitterionic and Neutral Arg Conformers

It is known that Arg forms stable zwitterions by intramolecular protonation of the guanidine group in aqueous solution.^3,34,35^ In our study, the neutral form and two zwitterionic forms of Arg (N, Z1, and Z2) were optimized. The Z1 structures were obtained from the neutral form by a single proton transfer from the carboxyl group to the guanidine group. On the other hand, the Z2 structure was formed with the α-amino group protonated rather than the guanidine side chain (Figure 1).^18^

Following the optimization with DFT methods in aqueous solution, it was observed that in the Z1 conformers, proton transfer occurred from the guanidinium group to the α-amino group, leading to the transition of the structure to the Z2 initial state (see Figures 7 and 1). The most stable structures both Z1W-1_[0.00]_^ωb97XD/6‑311++G(d,p)^ and Z1W-15_[0.00]_^B3LYP/6‑311++G(d,p)^ are characterized by two strong hydrogen bonds: C_ζ‑NH_-H···N–C_α‑NH_ and N_α‑NH_-H···O=C_α‑COOH_. Analysis with the ωB97XD/6-311++G­(d,p) method identifies the Z1W-1 as the most stable structural form, which appears with a frequency of 36.53%. Conversely, calculations using the B3LYP/6-311++G­(d,p) method reveal that Z1W-15 possesses a remarkably high abundance of 70.86%. This substantial prevalence indicates that Z1W-15 is a dominant structure within the system and may have a greater potential for interaction compared with other conformers. The attainment of such a high abundance by Z1W-15 underscores its critical importance in terms of stability and reactivity; thus, this conformer is regarded as a key element for understanding the overall behavior and interactions within the system (Table 6).

**Figure 7:**
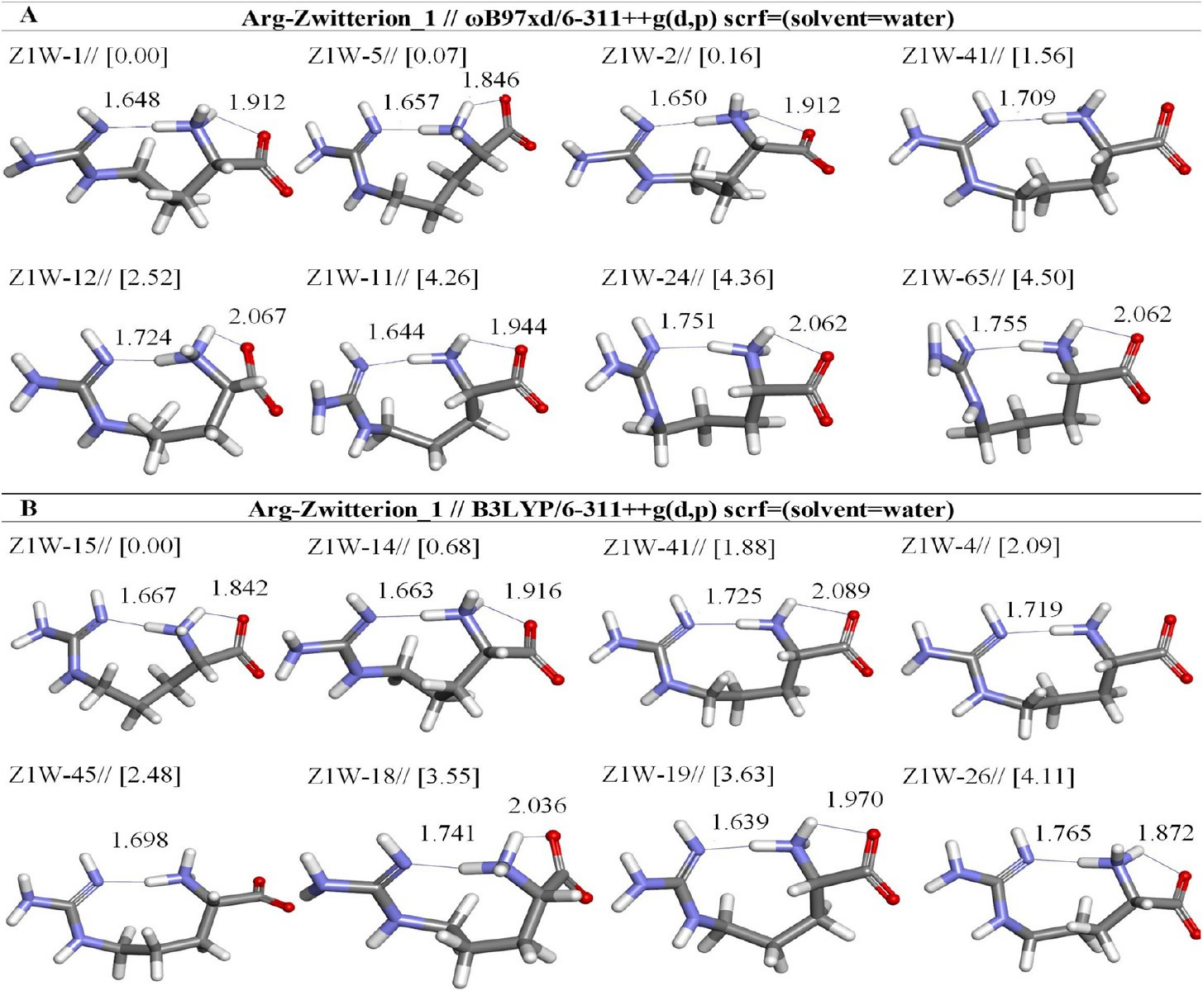
Optimized results of zwitterion Arg conformers (Z1) in aqueous by two different DFT methods at the (A) ωb97XD and (B) B3LYP/6-311++G** levels. Distances are given in Å.

**Table 6:** Calculated Total Electronic Energies (*E*_tot_, Hartree), Zero-Point Vibrational Energy Correction (*E* + ZPE, Hartree), Dipole Moment (Debye), and Relative Energies (Δ*E*_rel_, kcal/mol) Using the DFT Method at the B3LYP and ωb97XD Levels of Theory for Zwitterion (Z1) Species of Arginine

opt. conformer structure	*E*_tot_ (Hartree)^a^	μ (Debye)	E + ZPE (Hartree)^a^	Δ*E*_rel_ (kcal mol^–1^)	hydrogen bond	X···H^b^ distance (Å)	(%)
Arg-Zwitterion1-ωb97xd/6-311++g(d,p) (solvent = water)							
Z1W-1	–606.5820	17.67	–606.3562	0.00	:H7-:N21	1.648	36.53
					:H26-:O4	1.912	
Z1W-5	–606.5819	17.77	–606.3562	0.07	:H26-:N21	1.657	32.29
					:H5-:O4	1.846	
Z1W-2	–606.5818	17.34	–606.3559	0.16	:H5-:N21	1.650	27.98
					:H7-:O6	1.912	
Z1W-41	–606.5795	19.74	–606.3536	1.56	:H7-:N21	1.709	2.62
Z1W-12	–606.5780	15.91	–606.3517	2.52	:H5-:N21	1.724	0.52
					:H7-:O6	2.067	
Z1W-11	–606.5752	16.01	–606.3495	4.26	:H26-:N21	1.644	0.03
					:H7-:O4	1.944	
Z1W-24	–606.5751	16.96	–606.3485	4.36	:H5-:N21	1.751	0.02
					:H26-:O4	2.062	
Z1W-65	–606.5748	15.18	–606.3482	4.50	:H5-:N21	1.755	0.02
					:H26-:O4	2.062	
Arg-Zwitterion1-b3lyp/6-311++g(d,p) (solvent = water)							
Z1W-15	–606.7707	18.37	–606.5480	0.00	:H26-:N21	1.667	70.86
					:H5-:O4	1.842	
Z1W-14	–606.7696	15.22	–606.5469	0.68	:H26-:N21	1.663	22.67
					:H5-:O6	1.916	
Z1W-41	–606.7680	18.79	–606.5450	1.88	:H7-:N21	1.725	2.99
					:H5-:O4	2.089	
Z1W-4	–606.7676	19.09	–606.5447	2.09	:H7-:N21	1.719	2.08
Z1W-45	–606.7663	18.69	–606.5440	2.48	:H26-:N21	1.698	1.07
Z1W-18	–606.7656	14.55	–606.5423	3.55	:H5-:N21	1.741	0.18
					:H7-:O6	2.036	
Z1W-19	–606.7649	17.69	–606.5422	3.63	:H5-:N21	1.639	0.16
					:H26–O4	1.970	
Z1W-26	–606.7565	9.84	–606.5327	9.61	:H5-:N21	1.765	0.00
					:H7-:O6	1.872	

a Hartree = 627.503 kcal/mol.

b X proton (N or O).

Z2W-15 is the most stable Z2 structure for both the B3LYP and ωb97XD levels. Both geometries differ mainly with regard to the orientations of the guanidine group (Figure 8). In addition, Z2W-15 at ωb97XD involved one weaker hydrogen bond in which N(22)···H(25) interacts with one oxygen atom (O(4)=C_α‑COOH_). This bond is approximately 2.792 Å in length (Table 7). In particular, the Z2W-16 and Z2W-32 structures in ωb97XD are less stable than the lowest energy Z2W-15 by 2.63 and 2.86 kcal/mol, respectively. These structures are important because they change to neutral forms by a single proton transferred from α-amino to the carboxylate group without a conformational adjustment from initial structures. These structures do not have hydrogen bonds (Figure 8a).

**Figure 8:**
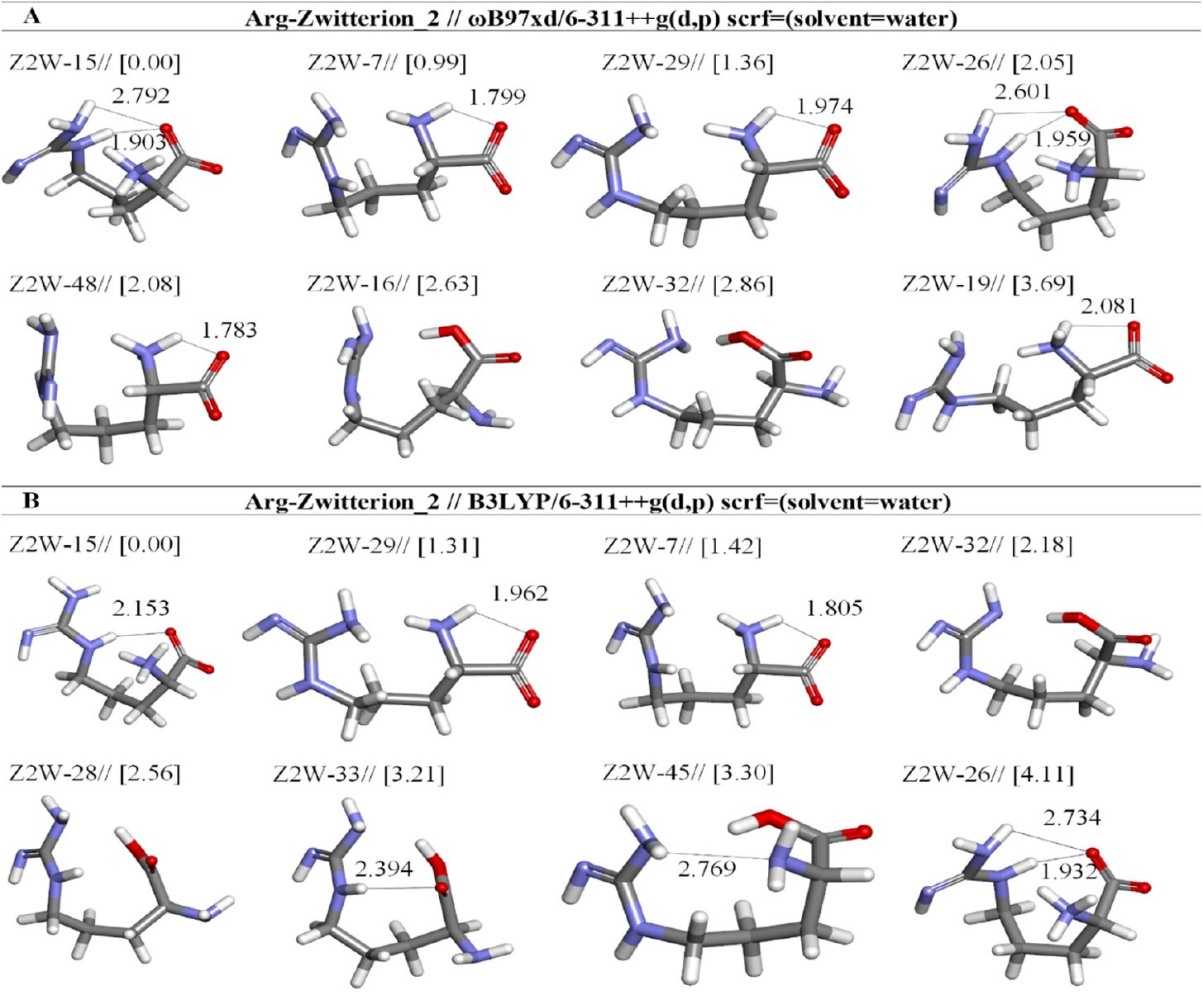
Optimized results of zwitterion Arg conformers (Z2) in aqueous by two different DFT methods at the (A) ωb97XD and (B) B3LYP/6-311++G** levels. Distances are given in Å.

**Table 7:** Calculated Total Electronic Energies (*E*_tot_, Hartree), Zero-Point Vibrational Energy Correction (*E* + ZPE, Hartree), Dipole Moment (Debye), and Relative Energies (Δ*E*_rel_, kcal/mol) Using the DFT Method at the B3LYP and ωb97XD Levels of Theory for Zwitterion (Z2) Species of Arginine

opt. conformer structure	*E*_tot_ (Hartree)^a^	μ (Debye)	*E* + ZPE (Hartree)^a^	Δ*E*_rel_ (kcal mol^–1^)	hydrogen bond	X···H^b^ distance (Å)	(%)
Arg-Zwitterion2-ωb97xd/6-311++g(d,p) (solvent = water)							
Z2W-15	–606.5715	9.60	–606.3426	0.00	:H25-:O4	2.792	73.02
					:H20-:O4	1.903	
Z2W-7	–606.5700	11.20	–606.3417	0.99	:H7-:O6	1.799	13.62
Z2W-29	–606.5694	12.25	–606.3417	1.36	:H7:-O4	1.974	7.31
Z2W-26	–606.5683	10.22	–606.3415	2.05	:H25-:O4	2.601	2.29
					:H20-:O4	1.959	
Z2W-48	–606.5682	17.31	–606.3411	2.08	:H7:-O4	1.783	2.17
Z2W-16	–606.5673	8.75	–606.3406	2.63			0.86
Z2W-32	–606.5670	7.54	–606.3406	2.86			0.59
Z2W-19	–606.5657	12.76	–606.3378	3.69	:H5-:O4	2.081	0.14
Arg-Zwitterion2-b3lyp/6-311++g(d,p) (solvent = water)							
Z2W-15	–606.7590	9.50	–606.5351	0.00	:H20-:O4	2.153	80.07
Z2W-29	–606.7576	12.00	–606.5330	1.31	:H7-:O4	1.962	8.83
Z2W-7	–606.7567	10.81	–606.5328	1.42	:H7-:O6	1.805	7.28
Z2W-32	–606.7548	7.53	–606.5316	2.18			2.01
Z2W-28	–606.7533	2.62	–606.5310	2.56			1.07
Z2W-33	–606.7521	2.92	–606.5299	3.21	:H20-:O6	2.394	0.36
Z2W-45	–606.7528	9.77	–606.5298	3.30	:H25-:N1	2.769	0.31
Z2W-26	–606.7529	10.19	–606.5285	4.11	:H25-:O4	2.734	0.08
					:H20-:O4	1.932	

a Hartree = 627.503 kcal/mol.

b X proton (N or O).

Z2W-26 is less stable than the first three Z2 structures described in Table 7. In particular, it is less stable than the lowest energy Z2W-15 by 2.05 kcal/mol, and they have a similar two hydrogen bonds. Furthermore, we have seen that the Z2W-15 and Z2W-26 that form open six-membered rings possess a chair-like conformation (Figure 4). On the B3LYP method, Z2W-32, Z2W-28, Z2W-33, and Z2W-45 returned to neutral form (Figure 8b). Looking at our optimization results, Z1 is predicted to be more stable than Z2 in both methods (Figure 9).

**Figure 9:**
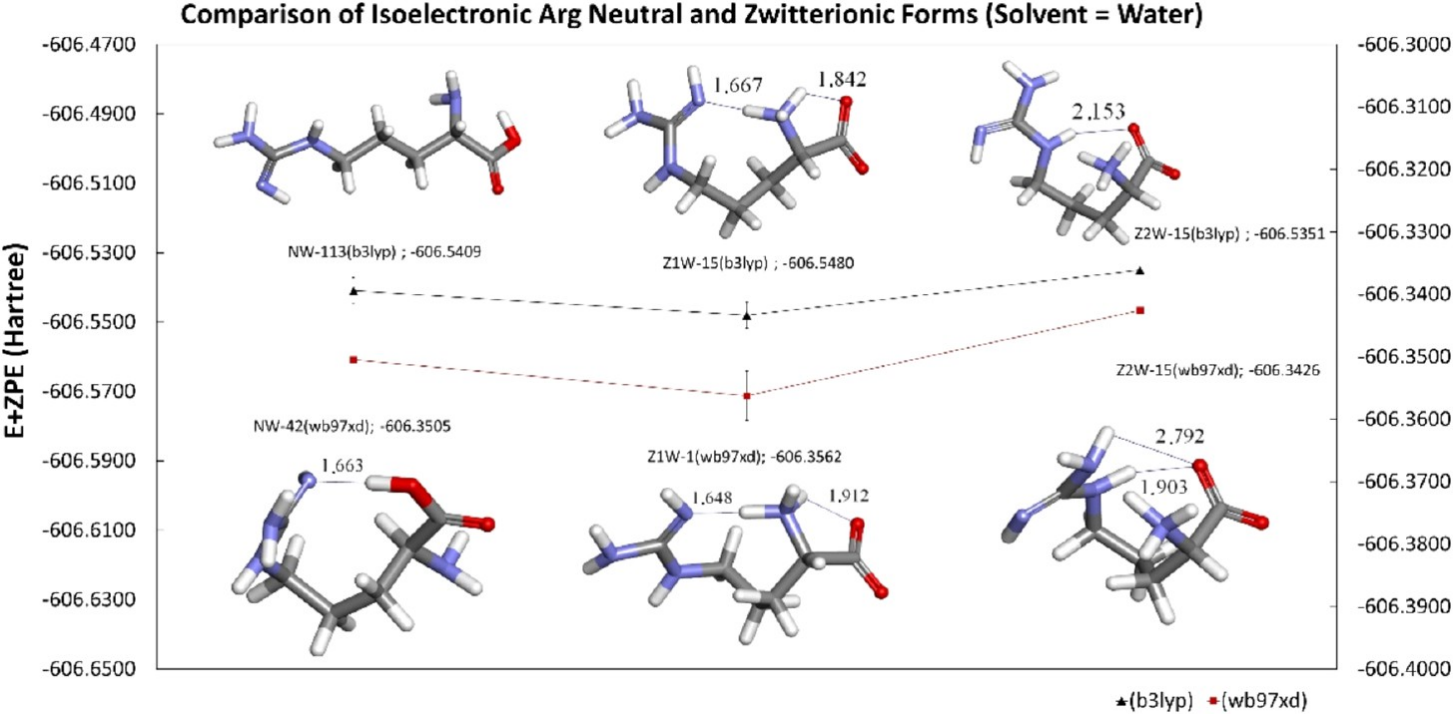
Comparison of isoelectronic Arg-neutral and zwitterionic forms in aqueous.

In the future, the protonation of guanidine groups may be a more accurate alternative than the protonation of α-amino groups for zwitterion structures. Utilizing the ωB97XD/6-311++G­(d,p) method, free energy calculations reveal that Z2W-15 stands out as the most stable conformer, demonstrating a significant prevalence of 73.02%. The subsequent conformer, Z2W-7, exhibits a notable presence of 13.62%. According to the results generated by the B3LYP/6-311++G­(d,p) approach, Z2W-15 shows the highest level of stability, with a representation of 80.07%. Following this, the Z2W-29 has an abundance of 8.83%, while the Z2W-7 shows an abundance of 7.28% (Table 7).

The highest-energy conformers among the neutral Arg conformers are NW-42 and NW-113, respectively, at the ωb97XD/6-311++G­(d,p) and B3LYP/6-311++G­(d,p) levels (Figure 10). We determined the geometric features of intramolecular hydrogen bonds for the highest-energy conformer structures. NW-42 is characterized by a strong H-bond (C_ζ‑NH_-N···HO-C_α‑COOH_) that is formed between the OH group and N(21), distance equals 1.663 Å, but NW-113 structure does not involve any hydrogen bond and NW-113 is more stable than NW-42 by 0.10 kcal mol^–1^ at the B3LYP level (Table 8). Although NW-42 was tailored for an aqueous setting, it closely resembles the neutral conformer (N2) outlined by Schlund et al. in the gas phase, as determined by the RI-MP2/TZVPP+ level of theory.^15,32^ However, this comparison should be considered qualitative as it involves different solution environments and computational levels. NW-411 is the eighth lowest energy structure, and it is 3.10 kcal/mol less stable than NW-42 according to the ωb97XD method. NW-411 is stabilized by a weak hydrogen bond formed between the guanidium group hydrogens and the carbonyl oxygen O(4) (Figure 10a). Similar structures to NW-411 have already been described in the literature for the gas phase.^15^ The results from free energy calculations using the ωB97XD/6-311++G­(d,p) and B3LYP/6-311++G­(d,p) methods indicate that the NW-42 and NW-113 are the most stable structures, with stability percentages of 76.59 and 22.45%, correspondingly (Table 8).

**Figure 10:**
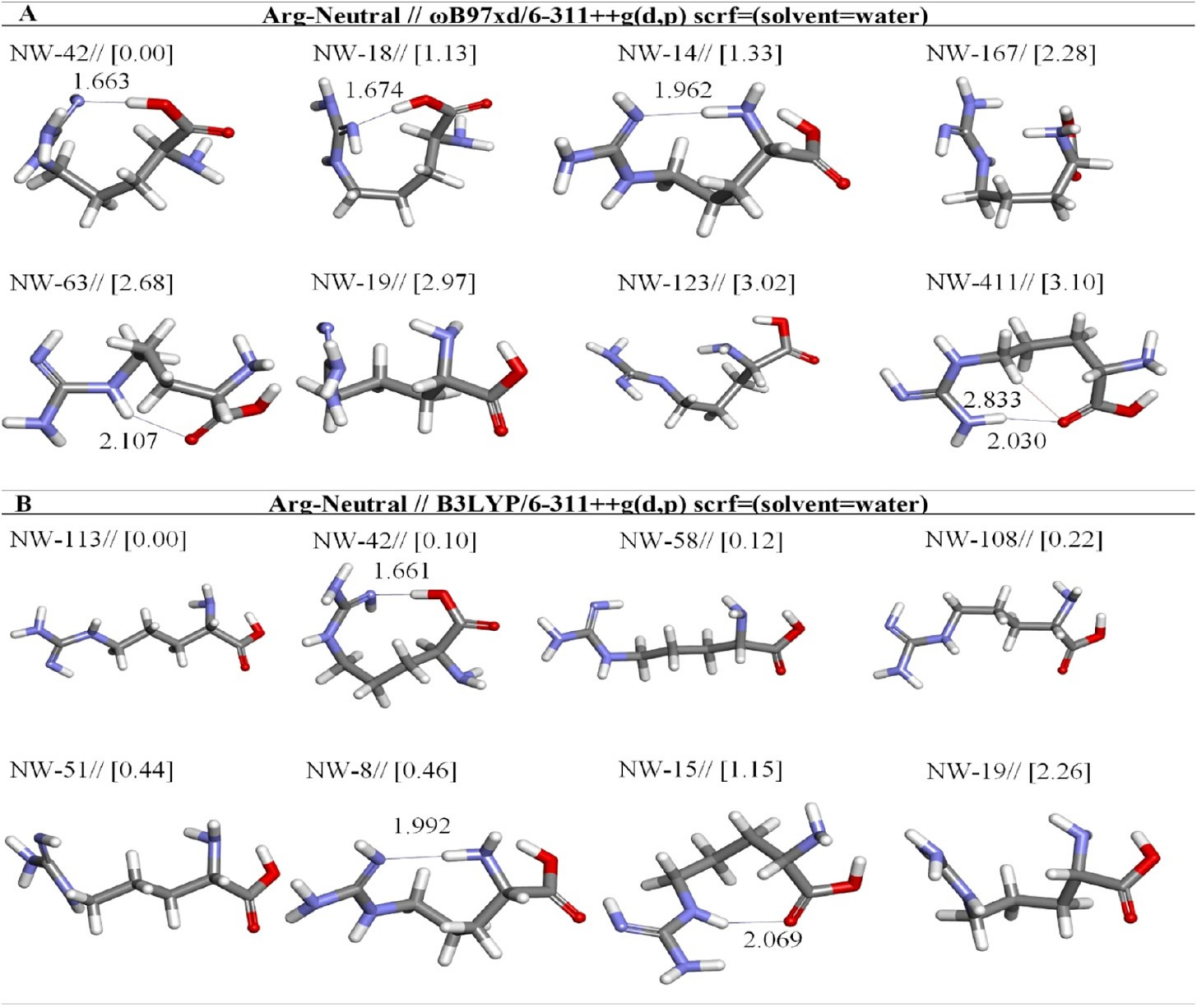
Optimized results of neutral Arg conformers in aqueous by two different DFT methods at the (A) ωb97XD and (B) B3LYP/6-311++G** levels. Distances are given in Å.

**Table 8:** Calculated Total Electronic Energies (*E*_tot_, Hartree), Zero-Point Vibrational Energy Correction (*E* + ZPE, Hartree), Dipole Moment (Debye), and Relative Energies (Δ*E*_rel_, kcal/mol) Using the DFT Method at the B3LYP and ωb97XD Levels of Theory for Neutral Species of Arginine

opt. conformer structure	*E*_tot_ (Hartree)^a^	μ (Debye)	*E* + ZPE (Hartree)^a^	Δ*E*_rel_ (kcal mol^–1^)	hydrogen bond	X···H^b^ distance (Å)	(%)
Arg-Neutral-ωb97xdd/6-311++g(d,p) (solvent = water)							
NW-42	–606.5759	9.17	–606.3505	0.00	:H26-:N21	1.663	76.59
NW-18	–606.5741	10.54	–606.3491	1.13	:H26-:N21	1.674	11.46
NW-14	–606.5738	10.08	–606.3483	1.33	:H7-:N21	1.962	8.09
NW-167	–606.5723	4.94	–606.3467	2.28			1.64
NW-63	–606.5716	7.79	–606.3462	2.68	:H20-:O4	2.107	0.83
NW-19	–606.5712	5.99	–606.3459	2.97			0.51
NW-123	–606.5711	4.55	–606.3459	3.02			0.47
NW-411	–606.5709	12.60	–606.3458	3.10	:H16-:O4	2.833	0.41
					:H24-:O4	2.030	
Arg-Neutral-b3lyp/6-311++g(d,p) (solvent = water)							
NW-113	–606.7624	8.85	–606.5409	0.00			22.45
NW-42	–606.7623	9.42	–606.5405	0.10	:H26-:N21	1.661	19.02
NW-58	–606.7622	5.69	–606.5406	0.12			18.23
NW-108	–606.7621	7.58	–606.5405	0.22			15.44
NW-51	–606.7617	3.46	–606.5401	0.44			10.75
NW-8	–606.7617	10.60	–606.5396	0.46	:H7-:N21	1.992	10.39
NW-15	–606.7606	9.03	–606.5384	1.15	:H20-:O4	2.069	3.22
NW-19	–606.7588	4.16	–606.5373	2.26			0.49

a Hartree = 627.503 kcal/mol.

b X acceptor (N or O).

In the aqueous solution, we observed that Z1 is more stable than both N and Z2, whereas Z2 is more stable than N according to both computational methods (Figure 9).

#### 3.1.4 Anionic Arg Conformers

The optimization results of the different anionic forms of Arg were described by using the DFT method in an aqueous solution. The low-energy structures of anionic Arg are displayed in Figure 11. The results indicated that the anionic form of the lowest energy conformers is the same in both B3LYP and ωb97XD. This most stable structure AW-4 is characterized by two hydrogen bonds at ωb97XD: the distances of the O(4)···H(24)­N(22) and the O(4)···H(20)­N(1) are 2.501 and 1.807 Å, respectively (Table 9). Nevertheless, based on the free energy calculations performed with the ωB97XD/6-311++G­(d,p) approach, AW-4 shows the greatest prevalence, accounting for 42.48% of the total. AW-244 is the second most stable structure, having an abundance of 40.38%, which is quite close to that of the most stable conformer (Table 9).

**Figure 11:**
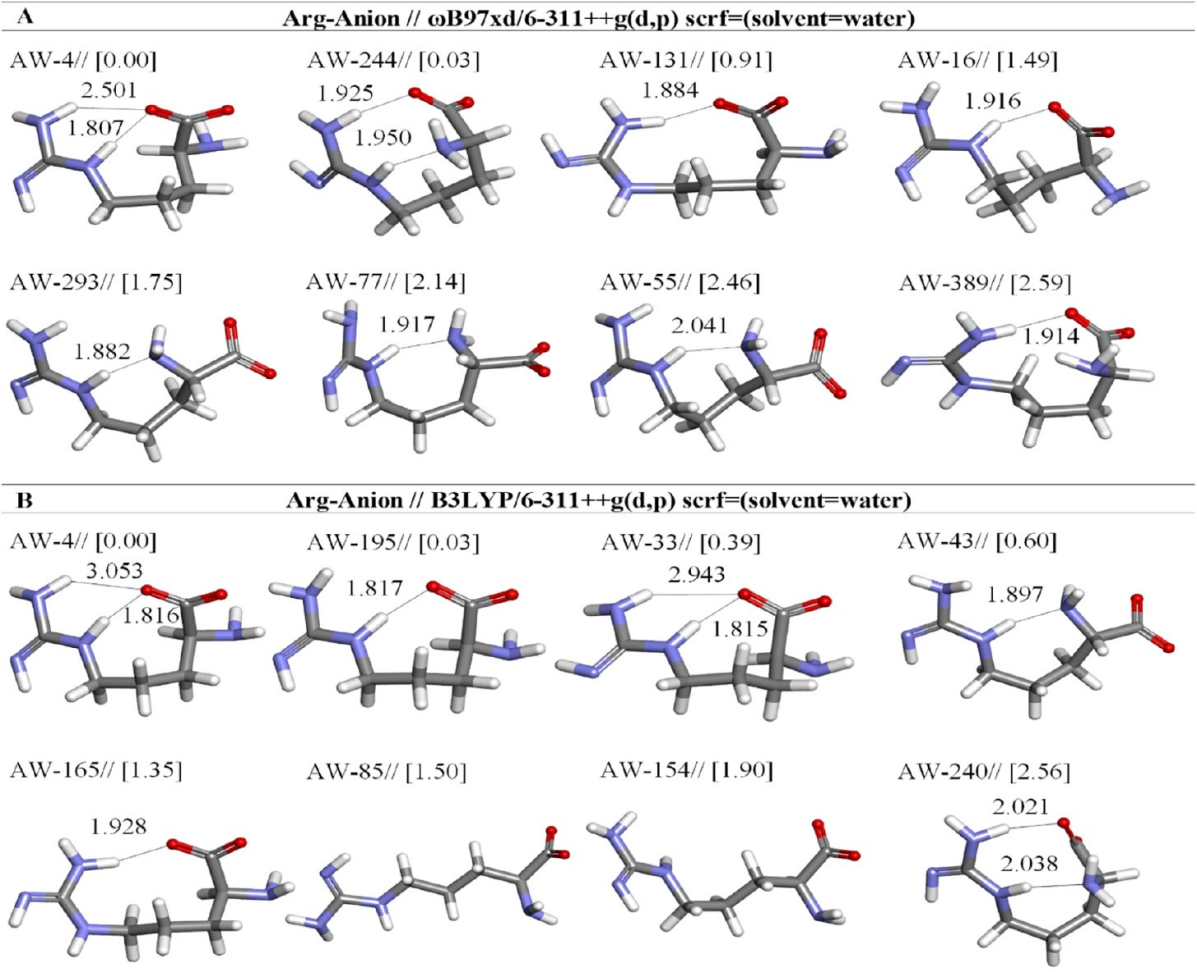
Optimized results of anionic Arg conformers in aqueous by two different DFT methods at the (A) ωb97XD and (B) B3LYP/6-311++G** level. Distances are given in Å.

**Table 9:** Calculated Total Electronic Energies (*E*_tot_, Hartree), Zero-Point Vibrational Energy Correction (*E* + ZPE, Hartree), Dipole Moment (Debye), and Relative Energies (Δ*E*_rel_, kcal/mol) Using the DFT Method at the B3LYP and ωb97XD Levels of Theory for Anionic Species of Arginine

opt. conformer structure	*E*_tot_ (Hartree)^a^	μ (Debye)	*E* + ZPE (Hartree)^a^	Δ*E*_rel_ (kcal mol^–1^)	hydrogen bond	X···H^b^ distance (Å)	(%)
Arg-Anion-ωb97xd/6-311++g(d,p) (solvent = water)							
AW-4	–606.1051	6.20	–605.8931	0.00	:H24-:O4	2.501	42.48
					:H20-:O4	1.807	
AW-244	–606.1051	7.32	–605.8925	0.03	:H25-:O6	1.925	40.38
					:H20-:N1	1.950	
AW-131	–606.1037	5.98	–605.8910	0.91	:H24-:O4	1.884	9.14
AW-16	–606.1028	5.83	–605.8906	1.49	:H20-:O6	1.916	3.43
AW-293	–606.1024	9.97	–605.8904	1.75	:H20-:N1	1.882	2.21
AW-77	–606.1017	13.17	–605.8897	2.14	:H20-:N1	1.917	1.15
AW-55	–606.1012	10.54	–605.8892	2.46	:H20-:N1	2.041	0.67
AW-389	–606.1010	8.10	–605.8889	2.59	:H25-:O6	1.914	0.54
Arg-Anion-b3lyp/6-311++g(d,p) (solvent = water)							
AW-4	–606.2975	6.34	–606.0889	0.00	:H24-:O4	3.053	32.55
					:H20-:O4	1.816	
AW-195	–606.2974	5.92	–606.0888	0.03	:H20-:O4	1.817	31.06
AW-33	–606.2967	5.95	–606.0882	0.39	:H24-:O4	2.943	16.89
					:H20-:O4	1.815	
AW-43	–606.2968	11.81	–606.0879	0.60	:H20-:N1	1.897	11.86
AW-165	–606.2959	9.98	–606.0867	1.35	:H24-:O4	1.928	3.32
AW-85	–606.2943	17.03	–606.0865	1.50			2.57
AW-154	–606.2938	12.84	–606.0858	1.90			1.31
AW-240	–606.2943	10.97	–606.0848	2.56	:H24-:O4	2.021	0.43
					:H20-:N1	2.038	

a Hartree = 627.503 kcal/mol.

b X acceptor (N or O).

When the same conformer structure was examined using the B3LYP method, it was observed that hydrogen bonds still formed between the same atoms but the bond lengths changed (Figure 11b). Hence these H-bonds are much weaker than the other (Figure 11). AW-33 is less stable than that of AW-4 by 0.39 kcal/mol at B3LYP. Also, it is seen that the geometry is the same as AW-4, and in the geometry of AW-4 and AW-33 revealed boat-like conformation (Figure 4).

Species AW-85 and AW-154 were characterized by using the B3LYP whereas AW-85 is closely related to the lowest energy AW-154 by only 0.40 kcal/mol. The nearly linear structures of AW-85 and AW-154 do not have any intramolecular H-bond (Figure 11b). The application of the B3LYP/6-311++G­(d,p) method reveals that AW-4 possesses a relative abundance of 32.55%, while AW-195 exhibits a slightly lower abundance of 31.06%, and AW-33 accounts for 16.89% of the total. AW-43 is noted at 11.86%, while the other conformers are listed with lower percentages (Table 9).

It has been observed that the structural charge properties of Arg anionic conformers are maintained in an aqueous solution.

### 3.2 Optimized Structure of Arginine in a Vacuum

All naturally occurring amino acids exist as zwitterions in aqueous solution over a wide range of pH. However, in the gas phase, the nonzwitterionic neutral forms are more stable. Therefore, when the nonzwitterionic structural forms of amino acids are employed in computational methods, vacuum conditions are significantly preferred over aqueous solutions. This preference stems from the complex interactions of zwitterionic structures in water and their sensitivity to various pH values. Consequently, when stability and energy calculations for amino acids are conducted, vacuum environments sometimes yield more reliable results. Arg, particularly, exists in a zwitterionic structure in aqueous solutions, whereas in the gas phase, its zwitterionic form can become more stable than the neutral form due to the presence of the positively charged guanidine side chain.^21,36,37^ However, the question of whether zwitterions of Arg exist as stable structures in the gas phase has been extensively discussed in the literature, and this article also focuses on this issue. The stability of Arg zwitterions in the gas phase can be influenced by various factors, including the physicochemical properties of this molecule and environmental conditions, making it a subject that requires a detailed examination.

#### 3.2.1 Zwitterionic and Neutral Arg Conformers

In this study, neutral and zwitterionic forms (N, Z1, and Z2) were identified by using the ωb97XD and B3LYP methods. When examining the zwitterionic forms after optimization with both DFT methods in gas phase, it was observed that in the Z1 conformers, the guanidium group loses its proton, transitioning to the Z2 initial structure (Figures 1 and 12). When analyzing the conformer structures in the Z2 group, it was noted that some low-energy structures lose the proton from the α-amino group, transitioning to the neutral form (Figures 1 and 13). This proton transfer contributes significantly to balancing the molecule’s charged properties, particularly in the Z1 group, aiding in the preservation of zwitterionic structures. Of particular interest, species of Z1V-1, Z1V-12, Z1V-24, and Z1V-41 were identified by using the ωb97XD method, whereas their structures are the same as the lowest energy zwitterionic structures in aqueous phase described before. Both Z1W-1 and Z1V-1 are stabilized by the same two hydrogen bonds in which N(1)­H(26) and N(1)­H(7) interact with oxygen atoms O(4) and N (21). The shorter bond is about 1.648 Å and the longer bond is about 1.912 Å. On the other hand, we found that the most stable structure was Z1V-1, and it is the same as the electronic energy and dipole moments described in Z1W-1 (Tables 6 and 10). So, we concluded that the most stable structure could be this zwitterionic form.

**Figure 12:**
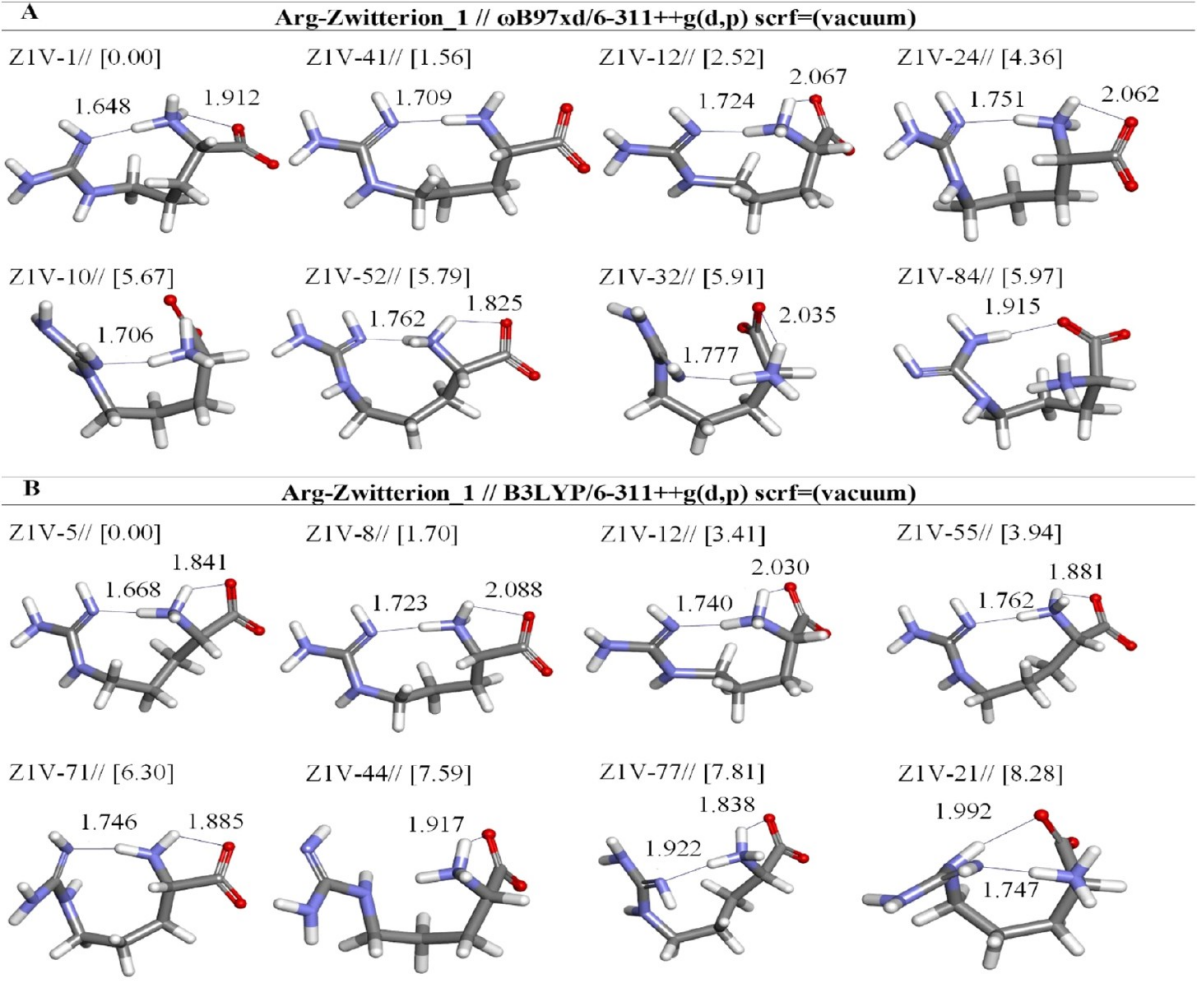
Optimized results of zwitterionic Arg (Z1) conformers in a vacuum by two different DFT methods at the (A) ωb97XD and (B) B3LYP/6-311++G** level. Distances are given in Å.

**Figure 13:**
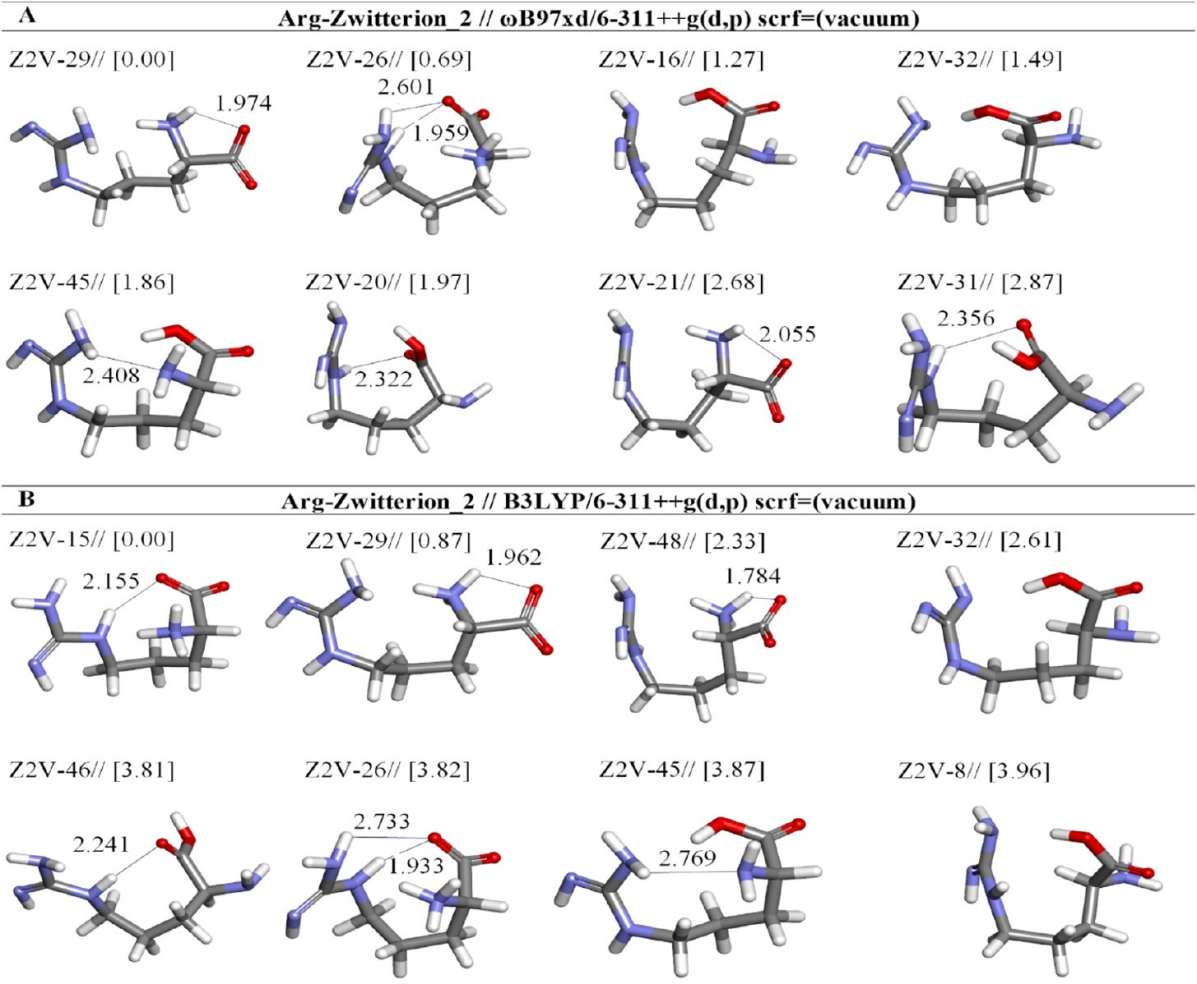
Optimized results of zwitterionic Arg (Z2) conformers in vacuum by two different DFT methods at the (A) ωb97XD and (B) B3LYP/6-311++G** levels. Distances are given in Å.

**Table 10:** Calculated Total Electronic Energies (*E*_tot_, Hartree), Zero-Point Vibrational Energy Correction (*E* + ZPE, Hartree), Dipole Moment (Debye), and Relative Energies (Δ*E*_rel_, kcal/mol) Using the DFT Method at the B3LYP and ωb97XD Levels of Theory for Zwitterionic (Z1) Species of Arginine

opt. conformer structure	*E*_tot_ (Hartree)^a^	μ (Debye)	*E* + ZPE (Hartree)^a^	Δ*E*_rel_ (kcal mol^–1^)	hydrogen bond	X···H^b^ distance (Å)	(%)
Arg-Zwitterion1-ωb97xd/6-311++g(d,p) (scrf = vacuum)							
Z1V-1	–606.5820	17.67	–606.3560	0.00	:H7-:N21	1.648	92.01
					:H26-:O4	1.912	
Z1V-41	–606.5795	19.74	–606.3536	1.56	:H7-:N21	1.709	6.60
Z1V-12	–606.5780	15.91	–606.3517	2.52	:H5-:N21	1.724	1.32
					:H7-:O6	2.067	
Z1V-24	–606.5751	16.96	–606.3485	4.36	:H5-:N21	1.751	0.06
					:H26-:O4	2.062	
Z1V-10	–606.5730	9.54	–606.3468	5.67	:H5-:N21	1.706	0.01
Z1V-52	–606.5728	19.81	–606.3466	5.79	:H7-:N21	1.762	0.01
					:H26-:O4	1.825	
Z1V-32	–606.5726	10.30	–606.3458	5.91	:H5-:N21	1.777	0.00
					:H7-:O6	2.035	
Z1V-84	–606.5725	10.53	–606.3439	5.97	:H24-:O4	1.915	0.00
Arg-Zwitterion1-b3lyp/6-311++g(d,p) (scrf = vacuum)							
Z1V-5	–606.7707	18.12	–606.5480	0.00	:H26-:N21	1.668	94.23
					:H5-:O4	1.841	
Z1V-8	–606.7680	19.55	–606.5450	1.70	:H7-:N21	1.723	5.35
					:H5-:O4	2.088	
Z1V-12	–606.7653	15.92	–606.5421	3.41	:H5-:N21	1.740	0.30
					:H7-:O6	2.030	
Z1V-55	–606.7644	17.40	–606.5412	3.94	:H7-:N21	1.762	0.12
					:H26-:O4	1.881	
Z1V-71	–606.7607	16.27	–606.5373	6.30	:H5-:N21	1.746	0.00
					:H26-:O4	1.885	
Z1V-44	–606.7586	12.85	–606.5347	7.59	:H26-:O6	1.917	0.00
Z1V-77	–606.7583	13.77	–606.5349	7.81	:H7-:N21	1.922	0.00
					:H26-:O4	1.838	
Z1V-21	–606.7575	14.84	–606.5336	8.28	:H20-:O6	1.992	0.00
					:H5-:N21	1.747	

a Hartree = 627.503 kcal/mol.

b X proton (N or O).

Z1V-41 is the second lowest energy structure in the gas phase, but it is the fourth lowest energy structure in aqueous solution. The obtained results indicated that it is less stable than Z1V-1 by only 1.56 kcal/mol. However, Z1V-41 has one relatively strong H-bond that can be identified between N(1)H and N(21). Z1V-84 is the eighth lowest energy structure, and it is characterized by only N_α‑NH_-H···O=C_α‑COOH_ H-bond (presented in Figure 12a). This weaker bond is formed between the H group and O(4) where the N(23)­H···O(4) distance equals 1.915 Å. The Z1V-5 identified by B3LYP levels is the lowest energy structure, and there are two strong hydrogen bonds in which N(1)H interacts with the oxygen atom (O4) and N (21) (Figure 12b). Moreover, it is more stable than Z1V-1.

At the B3LYP level of optimization, the energy differences among the Z1 structures are quite pronounced. For example, Z1V-21 is the eighth lowest energy structure by 8.28 kcal/mol (Table 10). Z1V-5 is stabilized by two weak H-bonds that can be identified as O(6)···HN(17) and N(21)···HN(1) distances are 1.992 and 1.747 Å, respectively. The B3LYP/6–311++G­(d,p) method indicates that Z1V-5 stands out as the most stable conformer, with a significant abundance of 94.23%. In contrast, the conformers Z1V-55 (0.12%), Z1V-71 (0.00%), Z1V-44 (0.00%), Z1V-77 (0.00%), and Z1V-21 (0.00%) display minimal abundances (Table 10).

The structures Z2V-15 and Z2V-29 have the lowest energies at the B3LYP and ωb97XD levels, respectively (Figure 13). But Z2V-15 is more stable than Z2V-29 by 0.87 kcal/mol (Table 11). Moreover, Z2V-15 differs from Z2V-29 in the location of the hydrogen bond. Z2V-15 and Z2V-29 are characterized by one hydrogen bond between N(17)­H···O(4) and N(1)­H···O(4) (Table 11). Moreover, some structures of Z2 are changed to neutral forms by a single proton transfer from α-amino to the carboxylate group. Hence, we found that the low-energy species which are labeled here as Z2 have similar structures to nonzwitterionic forms in the literature.^15,21,32^ We observed that the zwitterionic conformers of Z2V-16, Z2V-32, Z2V-45, Z2V-20, and Z2V-31 change to the less stable configuration of the neutral form at ωb97XD (Figure 13a). In all of these zwitterionic structures excluding Z2V-16 and Z2V-32, there are much weaker hydrogen bonds.

**Table 11:** Calculated Total Electronic Energies (*E*_tot_, Hartree), Zero-Point Vibrational Energy Correction (*E* + ZPE, Hartree), Dipole Moment (Debye), and Relative Energies (Δ*E*_rel_, kcal/mol) Using the DFT Method at the B3LYP and ωb97XD Levels of Theory for Zwitterionic (Z2) Species of Arginine

opt. conformer structure	*E*_tot_ (Hartree)^a^	μ (Debye)	*E* + ZPE (Hartree)^a^	Δ*E*_rel_ (kcal mol^–1^)	hydrogen bond	X···H^b^ distance (Å)	(%)
Arg-Zwitterion2-ωb97xd/6-311++g(d,p) (scrf = vacuum)							
Z2V-29	–606.5694	12.25	–606.3415	0.00	:H7-:O4	1.974	62.14
Z2V-26	–606.5683	10.22	–606.3403	0.69	:H25-:04	2.601	19.46
					:H20-:O4	1.959	
Z2V-16	–606.5673	8.75	–606.3417	1.27			7.30
Z2V-32	–606.5670	7.54	–606.3405	1.49			4.99
Z2V-45	–606.5664	9.78	–606.3406	1.86	:H25-:N1	2.408	2.68
Z2V-20	–606.5662	2.35	–606.3405	1.97	:H20-:O4	2.322	2.22
Z2V-21	–606.5651	15.93	–606.3370	2.68	:H7-:O6	2.055	0.68
Z2V-31	–606.5649	2.77	–606.3393	2.82	:H20-:O4	2.356	0.53
Arg-Zwitterion2-b3lyp/6-311++g(d,p) (scrf = vacuum)							
Z2V-15	–606.7590	9.50	–606.5350	0.00	:H20-:O4	2.155	78.78
Z2V-29	–606.7576	12.00	–606.5330	0.87	:H7-:O4	1.962	18.24
Z2V-48	–606.7552	17.17	–606.5313	2.33	:H7-:O4	1.784	1.55
Z2V-32	–606.7548	7.53	–606.5316	2.61			0.97
Z2V-46	–606.7529	5.28	–606.5309	3.81	:H20-:O4	2.241	0.13
Z2V-26	–606.7529	10.19	–606.5285	3.82	:H25-:O4	2.733	0.13
					:H20-:O4	1.933	
Z2V-45	–606.7528	9.77	–606.5298	3.87	:H25-:N1	2.769	0.11
Z2V-8	–606.7526	5.19	–606.5297	3.96			0.10

a Hartree = 627.503 kcal/mol.

b X proton (N or O).

The Z2V-21 is particularly interesting because it changed to the anionic form. It is less stable than Z2V-29 by 2.68 kcal/mol, and it has a similar H-bond with a distance as large as 2.055 Å.

In this study, when the molecular geometry of Z2 was optimized using the B3LYP method, it was observed that similar to the optimization results obtained with ωb97XD, Z2V-46, Z2V-45, Z2V-32, and Z2V-8 underwent a transition to the neutral form by losing the proton from the guanidium group. Z2V-8 is the lowest in terms of energy levels and is approximately 3.96 kcal/mol less stable than Z2V-15. Z2V-46 is the fifth lowest energy and is less stable than Z2V-15 by 3.81 kcal/mol. Z2V-46 has o-hydrogen bonds in which N(17)H interacts with O­(4). Z2V-8 is less stable than Z2V-46 and is not connected by a hydrogen bond. The importance of the Z2V-26 structure is that it can also exhibit a chair-like conformation (see Figure 4).^15^ Based on the ωB97XD/6-311++G­(d,p) method, Z2V-29 is the most stable (62.14%), followed by Z2V-26 (19.46%) and Z2V-16 (7.30%). On the other hand, the B3LYP/6-311++G­(d,p) method indicates Z2V-15 as the most stable (78.78%), with Z2V-29 at 18.24% and Z2V-48 at 1.55%. Other conformers show minimal abundance (Table 11).

Using DFT methods, we systematically explored all eight lowest-energy neutral structures, paralleling our approach to the zwitterionic forms. Among the lowest energy structures, NV-42 has a strong H-bond between N(21) and H–C_α‑COOH_ ^15^ (Table 12). Although NV-99 is the lowest energy and NV-42 is the second lowest energy by 0.10 kcal/mol at B3LYP, NV-42 is more stable than NV-99 (Figure 14). Furthermore, the structure of NV-99 is nearly linear and does not involve any H-bonds (see Figure 14b). Notably, unlike previous studies, our results indicate that neutral species, with the exception of NV-8, NV-42, NV-4, NV-1, and NV-15, do not exhibit intramolecular hydrogen bonds. The NV-42 is identified as the most stable according to the ωB97XD/6-311++G­(d,p) method, with a stability percentage of 55.38%. It is followed by NV-1 at 34.28% and NV-8 at 7.82%. Moreover, NV-99 is recognized as the most stable configuration by the B3LYP/6-311++G­(d,p) approach, showing a stability of 25.05%, while NV-42 (21.22%) and NV-108 (17.22%) have stability values (Table 12).

**Figure 14:**
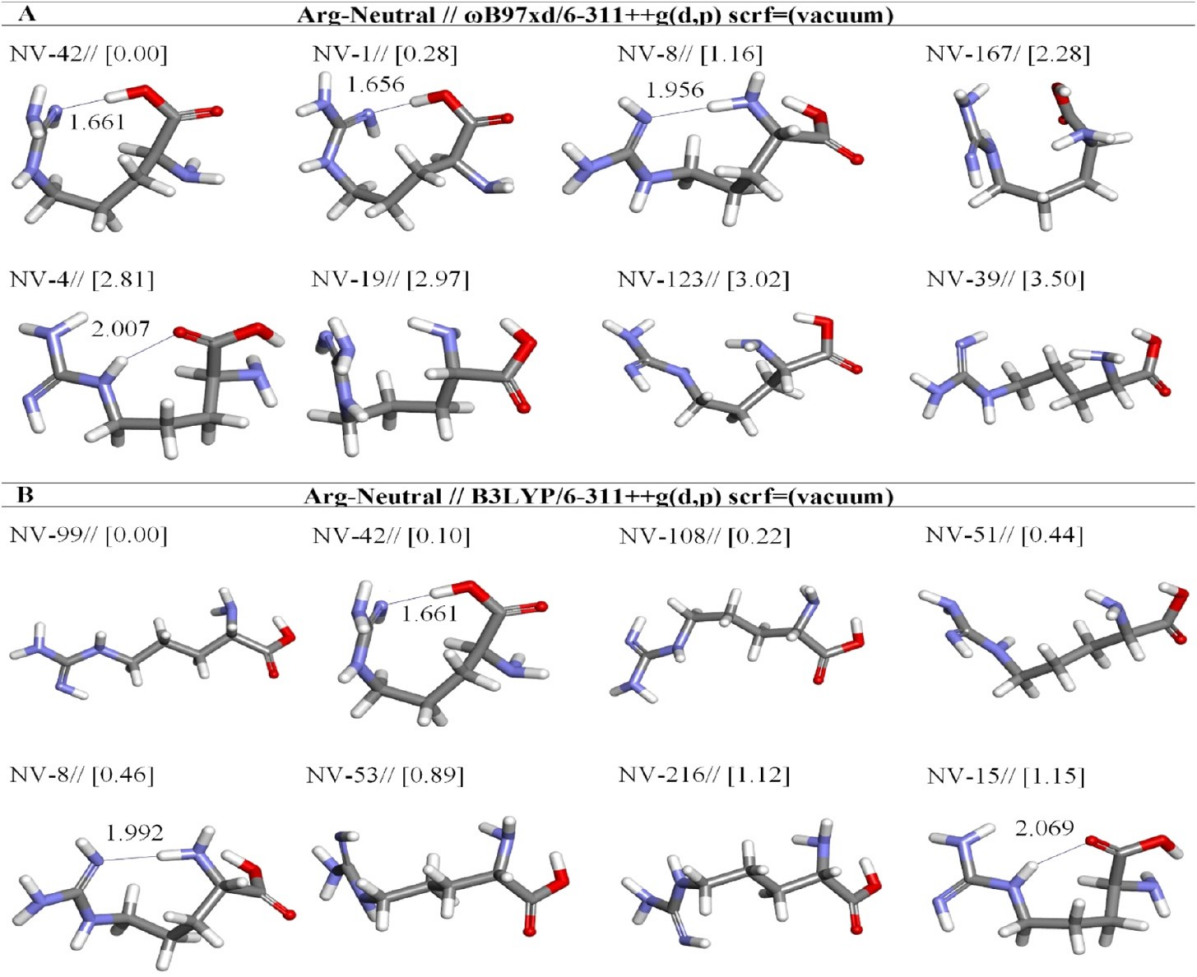
Optimized results of neutral Arg (N) conformers in vacuum by two different DFT methods at the (A) ωb97XD and (B) B3LYP/6-311++G** levels. Distances are given in Å.

**Table 12:** Calculated Total Electronic Energies (*E*_tot_, Hartree), Zero-Point Vibrational Energy Correction (*E* + ZPE, Hartree), Dipole Moment (debye), and Relative Energies (Δ*E*_rel_, kcal/mol) Using the DFT Method at the B3LYP and ωb97XD Levels of Theory for Neutral (N) Species of Arginine

opt. conformer structure	*E*_tot_ (Hartree)^a^	μ (Debye)	*E* + ZPE (Hartree)^a^	Δ*E*_rel_ (kcal mol^–1^)	hydrogen bond	X···H^b^ distance (Å)	(%)
Arg-Neutral-ωb97xd/6-311++g(d,p) (scrf = vacuum)							
NV-42	–606.5759	9.17	–606.3505	0.00	:H26-:N21	1.661	55.38
NV-1	–606.5754	10.23	–606.3503	0.28	:H26-:N21	1.656	34.28
NV-8	–606.5740	10.54	–606.3487	1.16	:H7-:N21	1.956	7.82
NV-167	–606.5723	4.94	–606.3467	2.28			1.18
NV-4	–606.5714	9.06	–606.3462	2.81	:H20-:O4	2.007	0.48
NV-19	–606.5712	5.99	–606.3455	2.97			0.37
NV-123	–606.5711	4.55	–606.3451	3.02			0.34
NV-39	–606.5703	6.43	–606.3453	3.50			0.15
Arg-Neutral-b3lyp/6-311++g(d,p) (scrf = vacuum)							
NV-99	–606.7624	8.84	–606.5409	0.00			25.05
NV-42	–606.7623	7.58	–606.5405	0.10	:H26-:N21	1.661	21.22
NV-108	–606.7621	3.46	–606.5405	0.22			17.22
NV-51	–606.7617	8.58	–606.5401	0.44			11.99
NV-8	–606.7617	10.60	–606.5396	0.46	:H7-:N21	1.992	11.59
NV-53	–606.7610	11.53	–606.5392	0.89			5.58
NV-216	–606.7606	12.20	–606.5394	1.12			3.76
NV-15	–606.7606	9.03	–606.5384	1.15	:H20-:O4	2.069	3.59

a Hartree = 627.503 kcal/mol.

b X proton (N or O).

We can see that Z1 is more stable than both N and Z2 in a vacuum the same as that observed in the aqueous solution (Figure 15). Moreover, the dipole moment of the lowest energy zwitterion Z1 is higher than that of the lowest energy N and Z2 at both DFT methods. Also, we found that the low-energy species which are labeled here as Z1, Z2, and N have similar structures reported in the literature.^15,18,21,32^

**Figure 15:**
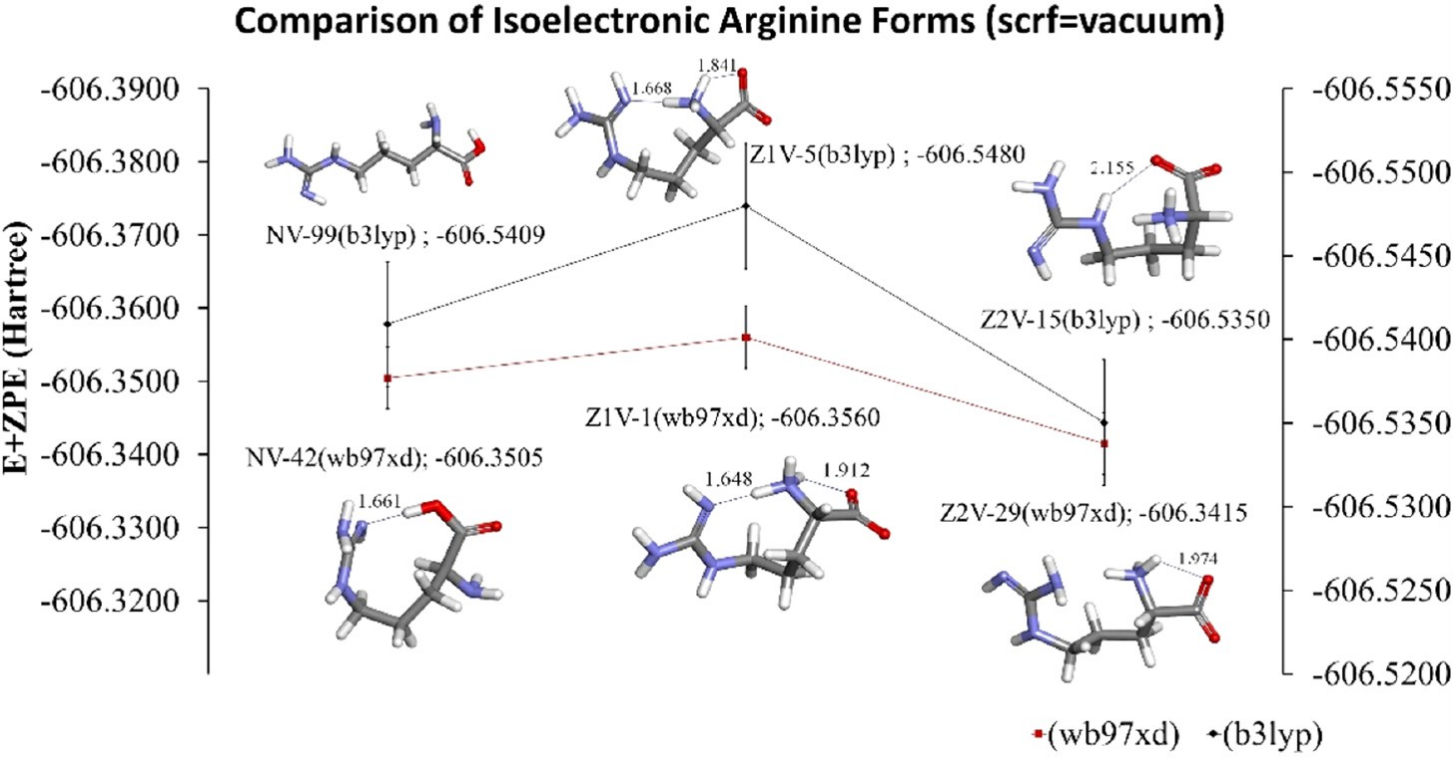
Comparison of isoelectronic Arg amino acids forms in a vacuum.

## 4 Conclusions

The results obtained using DFT methods exhibit a variety of stability rankings when we investigate eight different charge distributions of the Arg forms. The lowest energy conformers of eight Arg forms with different charge distributions were investigated at ωb97XD/6-311++G­(d,p) and B3LYP/6-311++G­(d,p) levels both in aqueous solution and in the gas phase.

We reported that the lowest zwitterionic structure Z1W is the most stable in electronic energy compared to the lowest neutral structure NW in both DFT methods. But Z2W structures were formed with the α-amino group protonated rather than the guanidine side chain; we have seen that Z2W might be converted to neutral form. Therefore, protonation of the guanidine group may appear to be a more favorable way to enhance the stability of Arg’s zwitterionic structure than protonation of the α-amino group. Protonation of the guanidine group can contribute to the stability of the molecular structure by maintaining the Arg zwitterionic structure. This can assist in balancing the charged properties of the Arg molecule and, consequently, in preserving its zwitterionic structure. Since similar results were obtained in a gas phase, this preference may be a general feature.

In this study, the two lowest-energy dicationic structures of Arg, DW-17, and DW-12, were identified for the first time using the B3LYP and ωb97XD methods, respectively. Furthermore, these structures were found to exhibit considerable stability. Interestingly, when it comes to the anion conformers, the lowest-energy structures were significantly less stable in an aqueous solution compared with other conformers. Additionally, our observations revealed that the lowest-energy structures of protonated Arg C3 were not stable in an aqueous environment and tended to convert into more stable neutral forms.^33^ But the lowest energy C3 is more stable than neutral forms, and their dipole moments are comparable to that of neutral and zwitterionic forms for both DFT methods and larger than that for the lowest energy structures of N and Z2.

In summary, the results indicate that amino acids can exhibit different stability profiles with varying charge distributions, emphasizing the complexity of charged structures at the molecular level. Additionally, this study highlights the effectiveness of density functional theory as a tool for the stability analysis of amino acid conformers.

## Supplementary Material



## Notes

The optimized geometries of the most stable Arg conformers obtained at the MP2/6-311++G­(d,p) level. (PDF)
