# Supplementary material for: Conformational Analysis of Neutral and Ionic Arginine Forms Using DFT Methods
Source: ACS Omega. 2025 Jun 20;10(25):26826–47. doi: 10.1021/acsomega.5c01697 (PMC12223864; doi:10.1021/acsomega.5c01697)
Supplement: Supplementary file 1 [file ao5c01697_si_001.pdf]

Title : Conformational Analysis of Neutral and Ionic Arginine Forms Using the DFT Methods

Authors: Fulya Çağlar [a*], Gözde Aksoy [b], Cenk Selçuki [a,b]

[a]Ege University; Graduate School of Health Sciences; Health Bioinformatics Programme

[b]Ege University; Faculty of Science, Department of Biochemistry

Corresponding Author

Fulya Çağlar

Ege University; Graduate School of Health Sciences; Health Bioinformatics Programme, 35040, Bornova, Izmir, Turkey

e-mail: 93190000151@ogrenci.ege.edu.tr or fulya_caglare@hotmail.com

ORCID: 0000-0001-6325-1980

Phone: +90-506-109 23 60

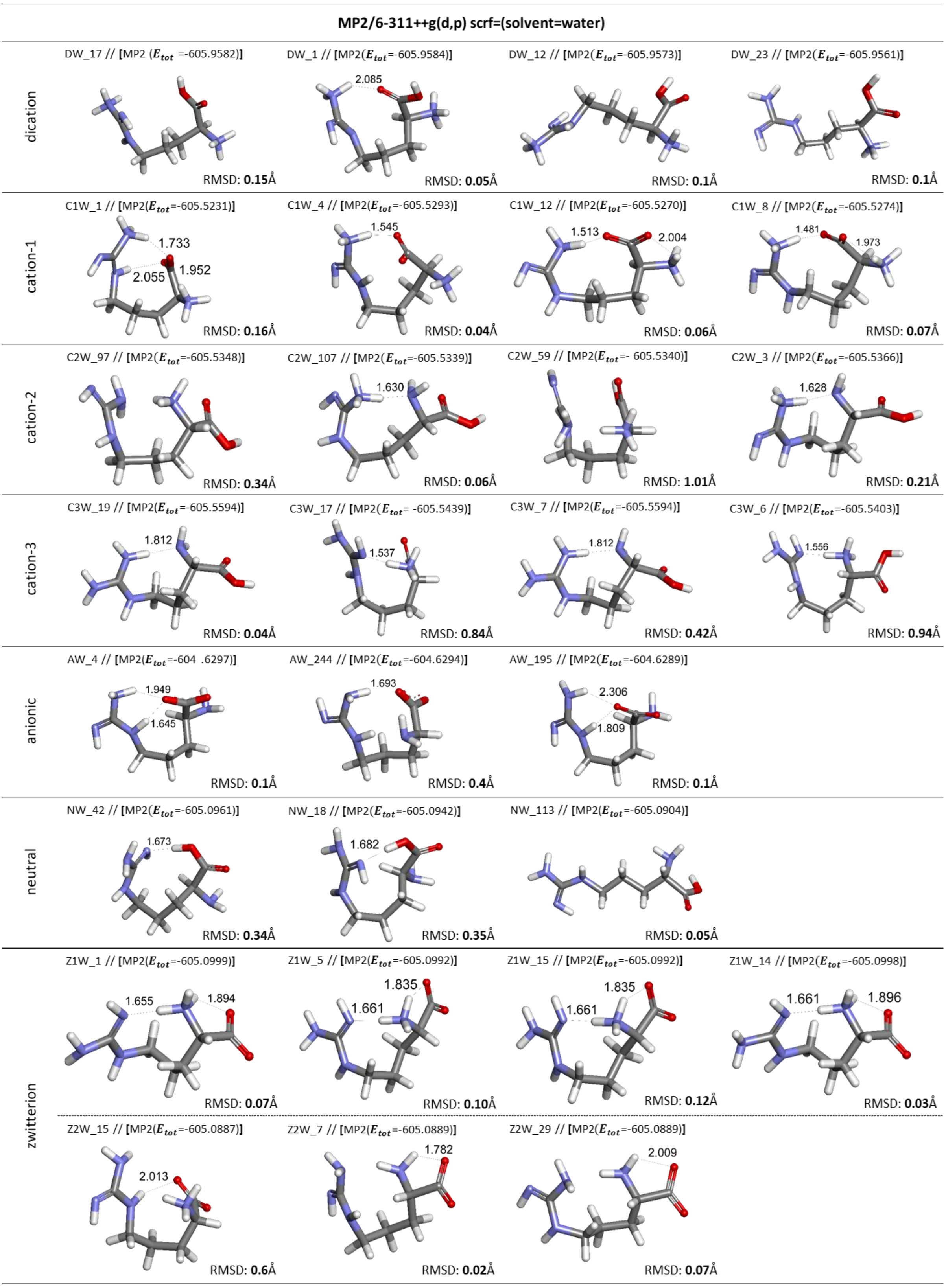

Figure S1. The structures of the most stable Arg conformers in aqueous have been optimized using the MP2/6-311++G(d,p) level. **E_{tot}** : Hartree.

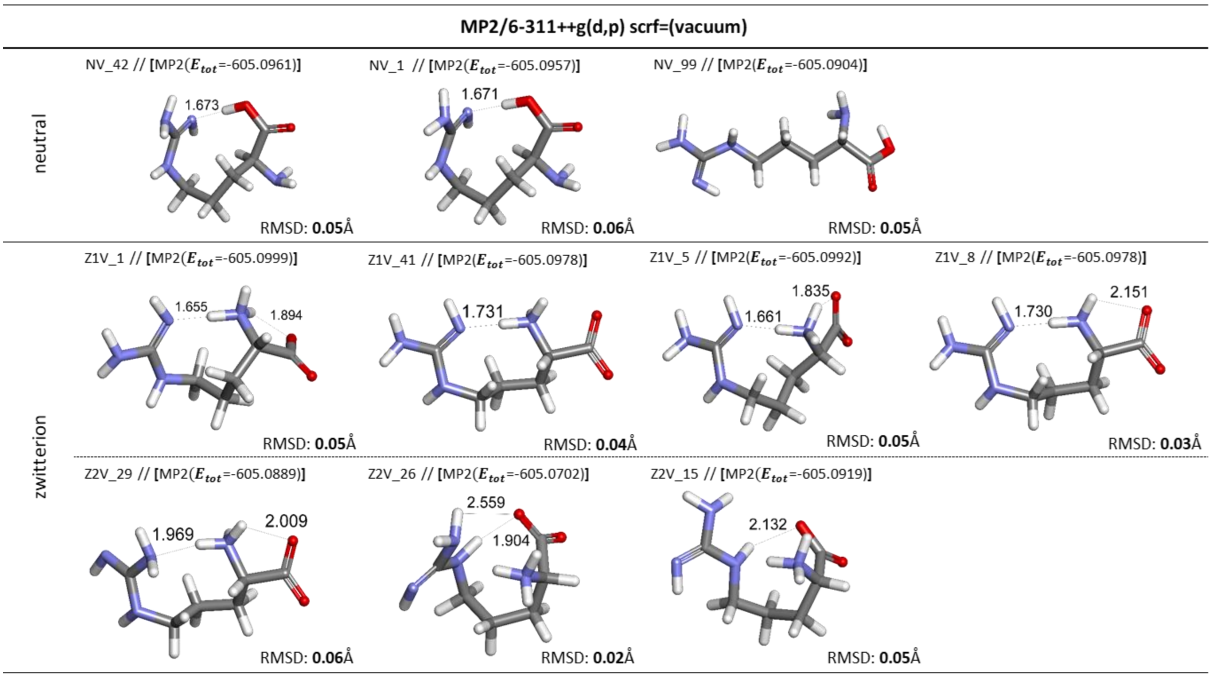

Figure S2. The structures of the most stable Arg conformers in vacuum have been optimized using the MP2/6-311++G(d,p) level. $\mathbf{E_{tot}}$: Hartree.
